# Production of a viral surface protein in *Nannochloropsis oceanica* for fish vaccination against infectious pancreatic necrosis virus

**DOI:** 10.1007/s00253-022-12106-7

**Published:** 2022-09-07

**Authors:** Sweta Suman Rout, Imke de Grahl, Xiaohong Yu, Sigrun Reumann

**Affiliations:** 1 Plant Biochemistry and Infection Biology, Institute of Plant Science and Microbiology, Universität Hamburg, Ohnhorststr. 18, 22609 Hamburg, Germany; 2 Zybio Inc, Chongqing Municipality, 400084 China

**Keywords:** Aquaculture, Flow cytometry, Infectious pancreatic necrosis, *Nannochloropsis oceanica*, Recombinant protein production, Subunit vaccine

## Abstract

**Abstract:**

*Nannochloropsis oceanica* is a unicellular oleaginous microalga of emerging biotechnological interest with a sequenced, annotated genome, available transcriptomic and proteomic data, and well-established basic molecular tools for genetic engineering. To establish *N. oceanica* as a eukaryotic host for recombinant protein synthesis and develop molecular technology for vaccine production, we chose the viral surface protein 2 (VP2) of a pathogenic fish virus that causes infectious pancreatic necrosis as a model vaccine. Upon stable nuclear transformation of *N. oceanica* strain CCMP1779 with the codon-optimized *VP2* gene, a Venus reporter fusion served to evaluate the strength of different endogenous promoters in transformant populations by qPCR and flow cytometry. The highest VP2 yields were achieved for the elongation factor promoter, with enhancer effects by its N-terminal leader sequence. Individual transformants differed in their production capability of reporter-free VP2 by orders of magnitude. When subjecting the best candidates to kinetic analyses of growth and VP2 production in photobioreactors, recombinant protein integrity was maintained until the early stationary growth phase, and a high yield of 4.4% VP2 of total soluble protein was achieved. The maximum yield correlated with multiple integrations of the expression vector into the nuclear genome. The results demonstrate that *N. oceanica* was successfully engineered to constitute a robust platform for high-level production of a model subunit vaccine. The molecular methodology established here can likely be adapted in a straightforward manner to the production of further vaccines in the same host, allowing their distribution to fish, vertebrates, or humans via a microalgae-containing diet.

**Key points:**

*• We engineered N. oceanica for recombinant protein production.*

*• The antigenic surface protein 2 of IPN virus could indeed be expressed in the host.*

*• A high yield of 4.4% VP2 of total soluble protein was achieved in N. oceanica.*

**Supplementary Information:**

The online version contains supplementary material available at 10.1007/s00253-022-12106-7.

## Introduction

Photoautotrophic algae are key organisms in marine ecosystems due to their high CO_2_ fixation capacity and as primary producers in the aquatic food chain (Sayre 2010). Unicellular microalgae possess enormous morphological, physiological, and genetic diversity, and only a small proportion of the estimated 1 million species has been identified and characterized in detail to date (Guiry 2012). Microalgae are also natural producers of biotechnologically relevant compounds of high value, including carotenoids, phycobilins, sterols, and health beneficial polyunsaturated fatty acids (PUFAs, reviewed by Borowitzka 2013). *Nannochloropsis* belongs to the class *Eustigmatophyceae* within the evolutionarily diverse group of heterokonts (stramenopiles) that evolved by secondary endosymbiosis of a red alga and a heterotrophic protist (Qiu et al. 2013). Interest in using *Nannochloropsis* for biotechnological applications is founded on its high content of omega-3 fatty acids (i.e., eicosapentaenoic acid), which reaches up to 4.3% (w/w) of biomass dry weight (BDW, Camacho-Rodríguez et al. 2014).

Additional high-value products that can possibly be produced in microalgae are recombinant proteins. Compared to prokaryotic expression systems, eukaryotic microalgae facilitate proper folding and correct posttranslational modifications of recombinant proteins (e.g., N- and O-glycosylation). Compared to plant and animal expression systems, microalgal growth rates are generally higher, and upscaling procedures are often easier and cheaper (Yan et al. 2016). In the past 5 years, both basic and advanced tools have been established for genetic engineering of *N. oceanica*, including efficient nuclear transformation techniques (Vieler et al. 2012), multipurpose expression vectors (Poliner et al. 2017; Zienkiewicz et al. 2017), and advanced methods for genome editing (Naduthodi et al. 2019; Poliner et al. 2018b). For recombinant protein production, the promoter is often the most decisive element (Sproles et al. 2021). Due to the large evolutionary distance even between species of the same genus, only endogenous promoters are generally functional in stramenopiles (Akbari et al. 2014; Schroda et al. 2000). In a screen for the most suitable promoters for the production of the fluorescent reporter protein Venus in *N. oceanica*, we selected those of elongation factor (EF) and nitrate reductase (NR; de Grahl et al. 2020). A relatively high Venus content of 4.9% of total soluble protein (TSP) was achieved if the reporter protein was extended by a so-called leader sequence (LS), i.e., the N-terminal NR peptide (de Grahl et al. 2020). However, despite significant progress, the production of only two recombinant proteins for biopharmaceutical applications has been reported in *Nannochloropsis* so far. The antimicrobial peptide bovine lactoferricin was produced in *N. oculata* at 4.3% of TSP using a heat inducible promoter from *Chlamydomonas reinhardtii* to protect Medaka (the fish *Oryzias latipes*) against gastrointestinal infection by the gram-negative bacterium *Vibrio parahaemolyticus* (Li and Tsai 2009). In *N. oculata*, the same expression elements allowed the production of a fish growth hormone at a considerable yield (0.42 mg/l culture), and the hormone indeed stimulated the growth of red tilapia larvae (Chen et al. 2008).

Microalgae including *Nannochloropsis* are particularly suitable for vaccine production for aquaculture because they are already part of the fish diet, serve to provide PUFAs and carotenoids, and can protect oral vaccines by bioencapsulation; hence, they may potentially be used for future oral fish vaccination (Criscuolo et al. 2019; Shah et al. 2017). Only a few eukaryotic microalgae, however, have successfully been engineered for recombinant vaccine production for aquaculture to date (Criscuolo et al. 2019). The VP28 protein of the white spot syndrome virus was produced upon nuclear transformation in *Dunaliella salina* and was capable of immunizing crayfish (Feng et al. 2014). The protein p57 of *Renibacterium salmoninarum*, the causative agent of bacterial kidney disease, was produced in *C. reinhardtii* chloroplasts and induced a specific immune response in salmonids fed dried algal biomass (Siripornadulsil et al. 2007). In *Nannochloropsis*, recombinant vaccine production has not been reported to date.

Viral diseases of humans, animals, and plants cause local or systemic infections with often severe and lethal courses. Farmed animals and aquaculture fish are prone to bacterial and viral infections, often leading to high mortality rates and economic losses (Charoonnart et al. 2018; Gomez-Casado et al. 2011). In the past few years, the worldwide demand for aquaculture fish has increased steadily (e.g., 80 million tons in 2017) to > 45% of consumed fish (FAO 2017). Infectious pancreatic necrosis (IPN) is a severe disease in salmon and trout and is caused by the IPN virus (Crane and Hyatt 2011). This pathogen infects particularly young fish and is responsible for severe disease outbreaks worldwide with high mortality (Biering et al. 2005; Dobos and Roberts 1983). Surviving fish individuals remain potent virus reservoirs for new disease outbreaks at any time (de las Heras et al. 2010). The IPN virus belongs to the genus *Aquabirnavirus* within the family *Birnaviridae* (Brown 1986). The genome of IPN virus consists of two double-stranded RNA molecules (Dobos 1995; Frost et al. 1995) that code for RNA-dependent RNA polymerase and a polyprotein that is autocleaved into two structural outer capsid proteins (VP2 and VP3) by the VP4 protease (Dobos 1995). The spikes are formed by VP2 and project radially outwards. One hypervariable loop (aa 206 to 350) in the surface-exposed domain of VP2 mediates host cell attachment and forms the major antigenic epitopes that are recognized by neutralizing antibodies (Coulibaly et al. 2010; Dopazo 2020; Frost et al. 1995; Lee et al. 2006), rendering the VP2 protein most suitable for vaccine production in yeast and insect cells against IPN virus (Allnutt et al. 2007).

In this study, we chose the VP2 protein of the IPN virus as a model vaccine to establish *N. oceanica* as a novel host for recombinant vaccine production in general and for future oral immunization of aquaculture fish in particular. To identify the optimal Venus-containing and reporter-free expression constructs, a complementary evaluation strategy was applied, combining expression analysis by qPCR with protein analyses by flow cytometry and immunoblotting. We started at the level of transformant populations to choose the best genetic elements and moved on to screening for the best overproducing individuals. Our analyses of VP2 yield and stability were extended to determining the number of integrated expression cassettes and revealed the presence of multiple expression cassettes in the best transformants of the highest VP2 productivity.

## Material and methods

### Microalgal strains and cultivation

*Nannochloropsis oceanica* CCMP1779 was received from the National Centre for Marine Algae and Microbiota (East Boothbay, USA) and cultivated in f/2 medium (Guillard and Ryther 1962), with increased nitrate and phosphate concentrations in batch cultures (1.8 mM NaNO_3_, 72 µM NaH_2_PO_4_) and photobioreactors (PBRs, 10 mM NaNO_3_, 0.4 mM NaH_2_PO_4_). For BDW determination, a glass-fiber filter (pore size 0.7 µm, 47 mm; VWR, Radnor, USA) was first dried at 105 °C for > 8 h and cooled down in a vacuum desiccator. For cell harvest and salt removal, 2*10^9^ cells (e.g., 15 ml of OD_540_ = 5) were washed (sedimentation at 4,000 × g, 10 min), resuspended in 50 ml H_2_O, and collected on the dried filter by vacuum filtration. The cells were dried (105 °C for > 8 h) and the BDW of the cells was determined (approx. 9 mg).

### Transformation vectors and transformation of *N. oceanica*

**Fig. 1:**
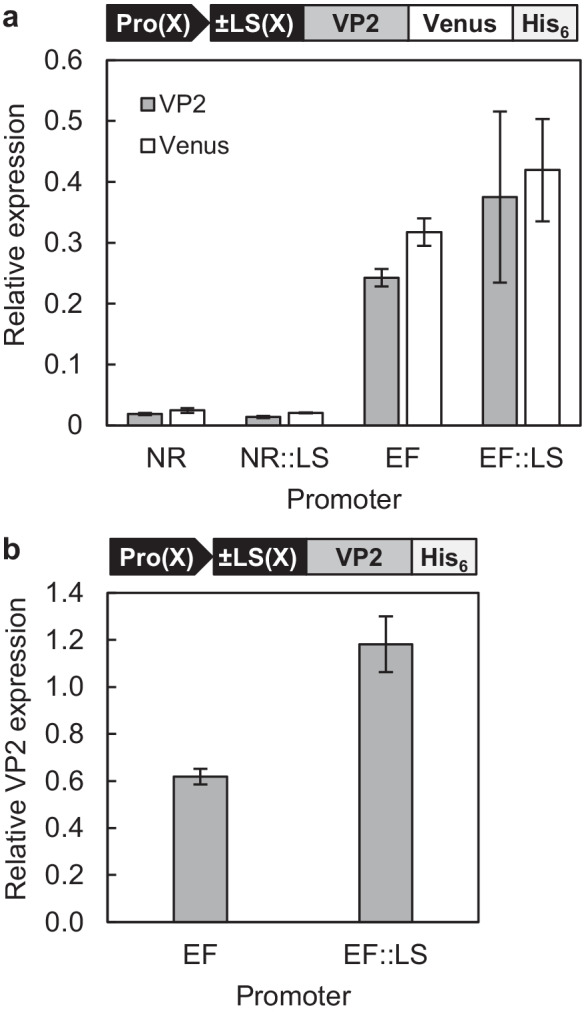
Comparative analysis of the strength of two endogenous promoters in populations of *N. oceanica* transformants. **a** Expression of the reporter gene fusion *VP2*-*Venus-His*_*6 *_was analyzed from the EF and NR promoters, either with or without the first 42 bp of their coding sequence (referred to as an LS). Relative expression levels of *VP2* and the *Venus* domain were determined by qRT–PCR and normalized to *ACT2*. **b** Expression of reporter-free *VP2-His*_*6*_ from the EF promoter (± LS) was determined likewise. Average values of two biological replicates with standard deviations are shown. Abbr.: Pro, promoter

The new expression plasmids for *N. oceanica* were based on our former plasmids and contained one expression cassette for the hygromycin resistance gene (*HygR*) and a second for *mVenus* as a reporter gene under the control of either the EF or the NR promoter (with or without LS, Figs. S11 and S12, Data S1; de Grahl et al. 2020). To generate the four Venus-tagged VP2 constructs [Pro(x)::(± LS(x))-VP2-Venus-His_6_; see Fig. 1a], the VP2 CDS (GenBank accession no. HQ457184, 1338 bp; Mutoloki and Evensen 2011) was codon-optimized (GenBank accession no. ON792393, Table S1, Eurofins Genomics; Ebersberg, Germany) and a *Mun*I restriction site was added to both ends. The *Mun*I excised *VP2* gene was cloned behind each promoter and upstream of the *Venus* reporter gene by the same site (Fig. 1a) according to standard protocols (Sambrook and Russell 2001). For the generation of the corresponding reporter-free *VP2* constructs (i.e., Pro(x)::(± LS(x))-VP2-His_6_; Fig. 1a), *VP2* was reamplified to add the nucleotides coding for a C-terminal His_6_-tag and a 5’ *Mlu*I restriction site (Table S2). Upon removal of *Venus* from the four previous overexpression vectors (de Grahl et al. 2020) by *Mlu*I and *Mun*I restriction, the new *VP2-His*_*6 *_amplicon was inserted. Stable nuclear transformants were generated by electroporation using linearized vector DNA (3 µg) as described previously (de Grahl et al. 2020). To verify the presence of the expression cassette in the transformants by PCR, genomic DNA of *N. oceanica* transformants was extracted according to a protocol modified from Edwards et al. (1991). From an *N. oceanica* culture (OD_540_ > 0.6), 2 ml was harvested and resuspended in 300 µl extraction buffer (200 mM Tris–HCl, pH 7.5, 250 mM NaCl, 25 mM EDTA, 0.5% (w/v) SDS). After cell lysis by heating (95 °C, 10 min) and sedimentation of cell debris (16,000 × g for 10 min), DNA was precipitated in 50% (v/v) isopropanol. The expression cassettes were identified using DreamTaq Green PCR Master Mix (Thermo Fisher Scientific™, Waltham, USA) and specific primer combinations (Table S2).

**Fig. 2:**
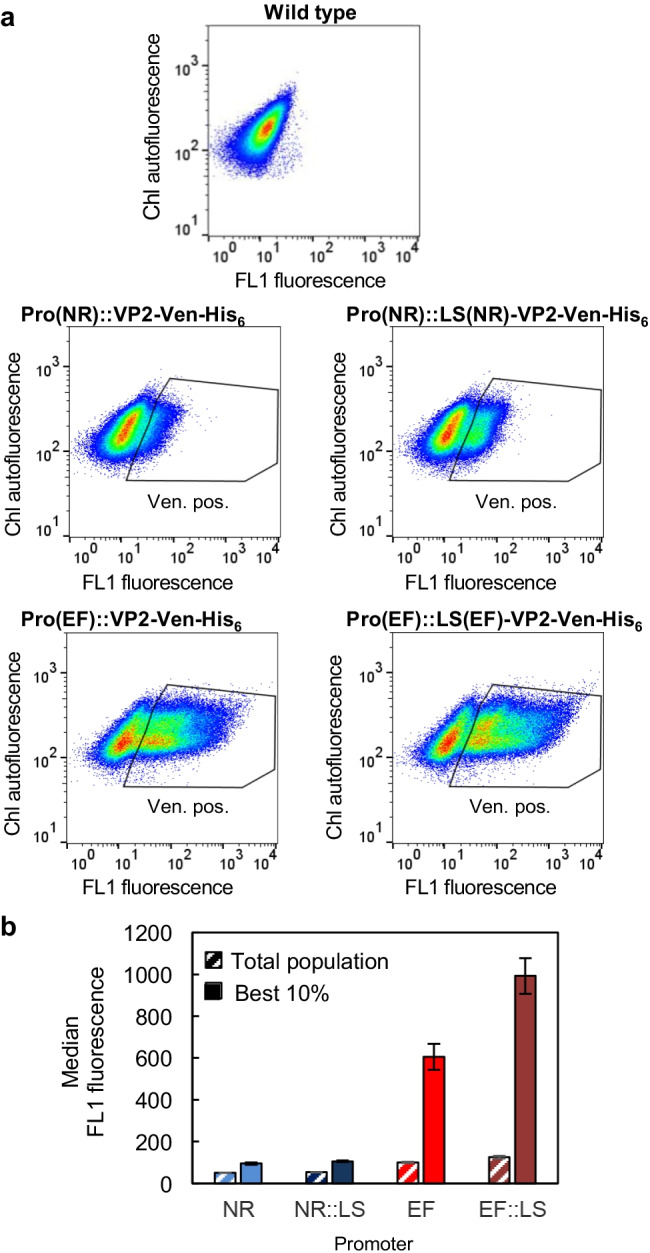
Quantification of VP2-Venus fluorescence in populations of *N. oceanica* transformants by flow cytometry. **a** Density plots of the wild-type strain and the transformant populations for measuring Venus fluorescence. The low level of FL1 fluorescence of the wild type was determined to gate for the Venus-positive (Ven.-pos.) cells of the transformant population as indicated. **b** For these selected Venus-positive cells, the median FL1 fluorescence was calculated either for the entire population (100%) or for the best 10% of transformants. One exemplary density plot of each population of two biological replicates is shown in **a**, and their average values with standard deviations are given in **b**

### Southern blot analysis

The hygromycin resistance gene (*HygR*) served as a probe (length: 858 bp) for Southern blotting. It was amplified by PCR (Table S2) and labeled using the digoxigenin High Prime DNA Labeling and Detection Starter Kit II (Roche Diagnostics GmbH, Mannheim, Germany). A *VP2*-specific probe (length: 1268 bp) was created similarly and served as a control. Total DNA of *N. oceanica* was isolated according to Varela-Álvarez et al. (2006), and 3 µg was digested with 30 units of *Hin*dIII or *Sac*I for 10 h and separated on a 0.8% (w/v) agarose gel. Digested plasmid DNA of pNoc ox Pro(EF)::VP2 (6 ng) and genomic DNA of the wild type served as positive and negative controls, respectively. After depurination, denaturation, and neutralization, the DNA was transferred to a positively charged nylon 6.6 membrane (Biodyne B Membrane, pore size 0.45 μm; Pall Life Sciences, Portsmouth, USA). The membrane was hybridized with the probe overnight at 52 °C. After a washing step, the membrane was blocked and incubated with polyclonal anti-digoxigenin antibodies conjugated to alkaline phosphatase, following the manufacturer’s instructions. Alkaline phosphatase was detected by the chemiluminescence reagent “CSPD ready-to-use” (Roche Diagnostics GmbH), using a CCD camera system (LAS-3000; Fujifilm, Tokyo, Japan).

### Expression analysis by qRT–PCR

*Venus* and *VP2* expression were analyzed in *N. oceanica* populations (> 40 individual transformants), as described previously (de Grahl et al. 2020). In brief, cells were grown individually in 48-well plates and pooled. RNA isolation of *N. oceanica* transformants and cDNA synthesis with an oligo(dT)_18_ primer were carried out as reported (de Grahl et al. 2020). Relative expression of *Venus* and/or *VP2* was determined with gene-specific primer pairs (Table S2) using actin2 (*ACT2*) as a reference gene (CCMP1779ǀ9086-mRNA-1; https://genome.jgi.doe.gov/Nanoce1779/). Quantification by qPCR was performed with FastStart Essential DNA Green Master Mix (Roche, Mannheim, Germany) and 1.5 µl of cDNA as a template using a LightCycler® 96 (Roche). The primer efficiencies were equally high for *Venus* (99%), *VP2* (98%), and *ACT2* (92%).

### Flow cytometry

Venus quantification was carried out in *N. oceanica* populations (> 40 individual transformants) by flow cytometry, as described above for qRT–PCR and previously (de Grahl et al. 2020). The cells were subjected to flow cytometry using an S3e cell sorter (Bio-Rad, Hercules, USA) equipped with a 488-nm excitation laser and detectors for side and forward scatter (i.e., SSC and FSC), FL1 (525/30-nm bandpass filter), and FL2 (560-nm longpass filter). Data were analyzed by the FlowJo software package (v10.6.1; BD Life Science, Ashlan, USA). To determine the median Venus fluorescence of the Venus-positive transformant populations or the background fluorescence of the wild-type cells, the following gating strategy was applied: *N. oceanica* cells were first gated based on their morphological features in a two-dimensional density plot of SSC-area versus FSC-area. Chlorophyll-positive cells were identified in a density plot of FL2-area versus FSC-area and displayed in a 3rd density plot (FL2-area versus FL1-area) to visualize the Venus-specific fluorescence of the transformant population.

### Protein isolation, immunoblotting, and mass spectrometry

The fraction of total soluble proteins (TSPs) of *N. oceanica* was isolated as described previously (de Grahl et al. 2020) and separated on 12.5% SDS–PAGE gels. Relative quantification of VP2-His_6_ (Figs. 4 and 5) was carried out by immunoblotting using a primary monoclonal mouse anti-His_6_ antibody (1:10,000 dilution, ab18184; Abcam, Cambridge, UK) and a secondary peroxidase-coupled anti-mouse antibody from rabbits. A pure His_6_-tagged standard protein (14 kDa) was used as a loading control to normalize the intensity of cross-reactivity. Chemiluminescence was quantified by ImageJ (de Grahl et al. 2020). To calculate the absolute VP2 content (Table 1), VP2-His_6_ was purified from one *N. oceanica* transformant (T3, + LS, day 23 of PBR1) under denaturing conditions in 8 M urea by Ni–NTA agarose (SERVA Electrophoresis GmbH, Heidelberg, Germany) to high homogeneity (Qiagen 2003, Fig. S8a). The purity (highest in the pooled eluates E2-E4, 86%) and the concentration of pure VP2-His_6_ were determined by Bradford assay (Bradford 1976) combined with SDS–PAGE, silver staining, and ImageJ analysis (Fig. S8a). The concentration of pure VP2 was precisely determined by calculating the mass of its silver-stained band based on a BSA calibration curve (25–150 ng, yielding c = 18 ng/µl pure VP2-His_6_, Fig. S8b). Subsequently, this purified VP2-His_6_ was used as a standard for absolute quantification of VP2-His_6_ in TSP fractions by immunoblotting (Table 1, Fig. S9). Blots were incubated sequentially with mouse monoclonal anti-VP2 (Fig. S9, 1:1,000 dilution, IBT systems GmbH, Binzwangen, Germany) or monoclonal anti-His_6_ (Fig. S10) and the same secondary antibody as mentioned above.

**Table 1:** VP2-His_6_ yield analyses of the best two *Nannochloropsis oceanica* transformants cultivated in small-scale PBRs. VP2 yields are given for day 20 (OD approx. 17) in the late linear growth phase (Fig. 5a). VP2 yields were determined by quantitative anti-VP2 immunoblotting and a calibration curve of pure VP2 (Figs. S8 and S9), and three technical replicates were averaged. The data were reproduced by a second biological replicate (PBR2) for both transformants and differed by < 0.1% of TSP

Transformant	VP2 yield			
	(% of TSP)	(% of T5 (− LS))	(mg*l^−1^)	(mg*g^−1^ BDW)
T5 (− LS)	1.4 ± 0.1	100	2.6 ± 0.2	1.5 ± 0.2
T3 (+ LS)	4.4 ± 0.3	310	5.7 ± 0.3	3.1 ± 0.2

In the TSP fraction, the identity of recombinant VP2-His_6_ was verified by mass spectrometry. A 50-kDa sample was cut out of the SDS–PAGE gel and the proteins were subjected to tryptic in-gel digestion (Shevchenko et al. 2006). Peptides were separated on a Dionex Ultimate 3000 UPLC system (Thermo Fisher Scientific, Waltham, USA) coupled to an Acclaim PepMap 100 C18 trap (Waters Corporation, Milford, USA) and a nanoEase m/z peptide BEH130 C18 column (Waters Corporation). A 60-min gradient with increasing acetonitrile concentration (2–30% (v/v)) was used. The eluted peptides were analyzed on a Quadrupole Orbitrap mass spectrometer (Q Exactive, Thermo Fisher Scientific). LC–MS/MS data were searched against the IPN virus protein database downloaded from UniProt (December 2020, 762 protein entries, https://www.ebi.ac.uk/UniProt/), against VP2-His_6_ and a protein database of common contaminants, using the Sequest algorithm integrated in Proteome Discoverer software version 2.4 (Thermo Fisher Scientific).

### Confocal and transmission electron microscopy

To estimate *Venus* expression in *N. oceanica* and verify cytosolic targeting, confocal microscopy was carried out with an inverted DMi8 microscope (Leica, Wetzlar, Germany) coupled to a confocal spinning disc unit (CSU X1; Yokogawa Electric Corporation, Musashino, Japan). Venus was excited with a 515-nm laser and light emission was detected with an appropriate filter (ET 535/30). Images were acquired by VisiView software (Visitron Systems, Puchheim, Germany). Confocal images were captured as single planes with a sCMOS camera system (QImaging OptiMOS, Digital Imaging Systems Ltd., Bourne End, UK).

For transmission electron microscopy, cells were sedimented by centrifugation and fixed with a mixture of 5 ml 1% (w/v) osmium tetroxide (in 0.1 M cacodylate buffer, pH 7.0), 2 ml 20 mM sucrose (in 8.3% (w/v) artificial seawater, ASW), and 800 µl 25% (v/v) glutaraldehyde by incubation on ice for 1.5 h (Karlson et al. 1996). After four washes with 8.3% (w/v) ASW, the cells were embedded in 2% (w/v) agarose (in 8.3% (w/v) ASW). After sample dehydration with a gradual increase in ethanol and infiltration in low viscosity Spurr resin (Spurr 1969), ultrathin sections of 70–80 nm were made with an ultramicrotome (Ultracut E, Leica-Reichert-Jung, Nußloch, Germany). Sections were viewed with a LEO 906 E transmission electron microscope (Zeiss, Jena, Germany) equipped with a MultiScan CCD camera (Model 794, Gatan, Munich, Germany). The images were processed by Digital Micrograph software (version 2.0.2., Gatan).

### Statistical analyses

For expression analyses of transformant populations by qRT–PCR, two biological replicates were analyzed to calculate the mean value and standard deviation. Each biological replicate was measured in three technical replicates of qRT–PCR (Fig. 1). For Venus fluorescence analyses by flow cytometry, the median FL1 fluorescence of the Venus-positive population was determined from two biological replicates and their mean value and standard deviation were calculated. The immunoblotting analyses were carried out for two biological replicates, and the mean values and standard deviations were determined for two (Figs. 4b and 5b) or three technical replicates (Table 1).

## Results

### Comparative promoter strength analyses of VP2-Venus fusions in transformant populations

To establish molecular technology for vaccine production in *N. oceanica*, we chose the major capsid protein of the IPN virus, VP2, as a model vaccine. The protein forms most of the antigenic epitopes that are recognized by neutralizing antibodies upon fish infection (Coulibaly et al. 2010; Dopazo 2020; Frost et al. 1995; Lee et al. 2006). The coding sequence of VP2 was codon-optimized for *N. oceanica* by generating a codon usage table deduced from 75 highly expressed genes included in an RNAseq dataset of *N. oceanica* CCMP1779 (Table S1, Vieler et al. 2012). To select the best endogenous promoter for *VP2* expression, we focused on two of six candidate promoters that had previously shown the highest strength in *Venus* expression in *N. oceanica* (de Grahl et al. 2020), namely, those of EF and NR. Both promoters were analyzed in combination with and without the 14-aa long N-terminal peptide of their own coding sequence, which was added N-terminally to VP2 as an LS (Fig. 1a; de Grahl et al. 2020). All constructs used the terminator of the lipid droplet surface protein of *N. oceanica* (Zienkiewicz et al. 2017). Upon transformation of *N. oceanica*, the expression vector presumably integrates randomly into the nuclear genome by double-strand breaks and nonhomologous end-joining, and stable transformants that are resistant to hygromycin can be selected (Zienkiewicz et al. 2017).

Random genome integration often leads to positional effects that alter transgene expression from the same promoter in different transformants (Schroda 2019). Therefore, we first tagged the VP2 C-terminally with the well-detectable yellow fluorophore, monomeric Venus, to average the expression strength of each cassette in transformant populations. Fusions of VP2-Venus were generated by subcloning *VP2* upstream of the *Venus* reporter gene in the four pNoc ox expression vectors generated previously (Table S2). Upon *N. oceanica* transformation by electroporation and after several selection rounds with hygromycin, 80–100 independent stable transformants were obtained. To verify the presence of the VP2-Venus expression cassette, total DNA was isolated from representative individual transformants and analyzed by genotyping PCR (Fig. S1, Table S2). To investigate heterologous gene expression from the four different promoter constructs and to verify that *VP2-Venus* was expressed at full length, both domains were quantified individually by qRT–PCR and their expression was normalized to the housekeeping reference gene, *ACT2* (Cao et al. 2012). All transformants were grown under standard growth conditions using nitrate as the sole nitrogen source rather than ammonium to ensure transgene expression from the nitrate-inducible NR promoter. Contrary to our previous data (de Grahl et al. 2020), the relative expression strength of *VP2* and *Venus* from the NR promoter was unexpectedly low (1.4–2.5%) relative to *ACT2* when averaged over the given transformant population (Fig. 1a). Hence, the NR promoter was not suitable for *VP2* gene expression. However, if expressed from the EF promoter, high transcript levels of *VP2-Venus* were reached, which corresponded to 30–40% of *ACT2* expression in the respective transformant populations (Fig. 1a).

Transcript and protein levels often but not always correlate (e.g., Richter et al. 2018). To quantify the reporter protein concentration, we applied flow cytometry to determine the cellular Venus fluorescence intensity in the same four transformant populations (Fig. 2). In these populations of transformants or the wild type, the level of chlorophyll autofluorescence was detected with the FL2 channel as an indicator of cell viability, and Venus fluorescence was quantified with the FL1 channel (Fig. 2a). The low background level of FL1 fluorescence of the wild type supported the specificity in detecting Venus fluorescence next to chlorophyll autofluorescence. Venus fluorescence was determined to identify and gate for Venus-positive cells (Fig. 2a). Within the transformant population carrying the NR promoter, only 12% (without LS) and 27% (with LS) of the cells were Venus positive (Fig. 2a, Fig. S2), which was consistent with the low transcript levels of *VP2-Venus* (Fig. 1a). In contrast, more than 55% of the EF promoter population (± LS) was Venus-positive with a bimodal distribution spanning four orders of magnitude, indicating a very high degree of heterogeneity of the transformants in Venus protein levels (Fig. 2a, Fig. S2). Accordingly, the median fluorescence of the Venus-positive populations of the EF promoter (+ / − LS) was > twofold higher than that of the NR promoter populations. When considering the entire population of Venus-positive cells, the median Venus fluorescence intensities of the two EF promoter populations differed only slightly (− LS: 100; + LS: 126, Fig. 2b). Additionally, the median Venus fluorescence of the 10% of cells possessing the highest Venus fluorescence was calculated by selecting the respective subpopulation from the density blots. This analysis showed a pronounced stimulatory effect of the LS on recombinant protein production, leading to an increase of 60% (− LS: 606; + LS: 993, Fig. 2b). According to confocal microscopy of individual EF promoter transformants, the VP2-Venus fluorescence was evenly distributed within the small cells except for the region of the single chloroplast and was concluded to be cytosolic (Fig. S3). Taken together, the EF promoter caused the highest level of *VP2-Venus* expression and Venus fluorescence, and the LS showed an enhancer effect on protein synthesis.

### Analyses of reporter-free VP2 constructs and selection of individual transformants

We next omitted the fluorophore gene and expressed the reporter-free *VP2* gene from the EF promoter (± LS). The *Venus* gene of the original pNoc ox expression vectors (de Grahl et al. 2020; Zienkiewicz et al. 2017) was replaced by the *VP2* sequence and extended by a C-terminal His_6_-tag to allow VP2 quantification by immunoblotting and optional protein purification by affinity chromatography. Stable *N. oceanica* transformants were generated and representative individual transformants were genotyped with vector-specific primer pairs, confirming the presence of the correct transgene expression cassette in the transformants (Fig. S1c, Table S2). In defined transformant populations, *VP2* expression was quantified by qRT–PCR, as described above. Interestingly, the *VP2* transcript levels were approximately twofold (− LS) and threefold (+ LS) higher than the levels of VP2-Venus, and they corresponded to 60% (− LS) and 120% (+ LS) of *ACT2* expression (Fig. 1b).

**Fig. 3:**
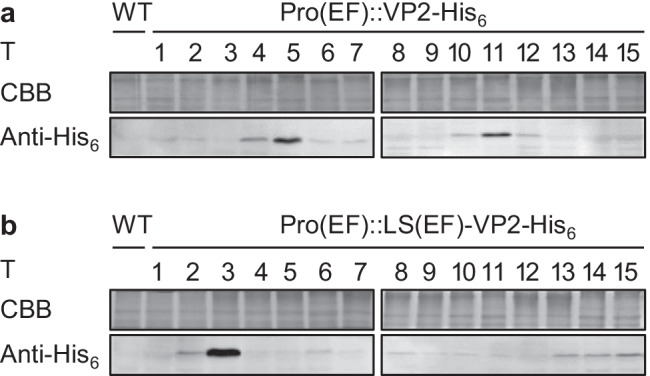
Immunoblotting screen for the best individual *N. oceanica* transformants overproducing reporter-free VP2-His_6_. **a** Upon *VP2* expression from the EF promoter without LS, the relative VP2 content (M = 50 kDa) of 15 individual transformants (T) was analyzed by immunoblotting and was the highest for transformants T5 and T11. **b** For the LS-containing construct (M = 51 kDa), transformant T3 showed an extraordinarily high VP2 content. The anti-His_6_ antibodies were specific for the blot section shown. Constant amounts of protein were loaded for immunoblotting (20 µg of TSP/lane), as verified by a separate gel stained by Coomassie Brilliant Blue (CBB, 10 µg/lane, region of 70–100 kDa shown). All immunoblots were done in parallel

The high variability of VP2-Venus fluorescence from the EF promoter in transformant populations (Fig. 2) suggested that the expression level was strongly determined by positional effects of the genomic integration site of the expression vector. We therefore next screened for the best individual transformants expressing reporter-free *VP2*. For each EF promoter construct, 15 individual transformants synthesizing (± LS)-VP2-His_6_ were analyzed for normal growth in 96-well plates and were subjected to upscaling in 100-ml batch cultures. In the mid-exponential growth phase (OD_540_ = 1.0–1.3), cells were harvested, TSPs were extracted, and the content of recombinant protein was analyzed by semiquantitative immunoblotting detecting the C-terminal hexahistidine tag. A specific immunoreactive band of approximately 50 kDa was detectable in most transformants and corresponded to the calculated molecular mass of (± LS)-VP2-His_6_ (50–51 kDa, Fig. 3). To verify the protein identity with VP2-His_6_ in one representative transformant, a slice of this region was excised from the SDS–PAGE gel and subjected to tryptic digestion followed by peptide analysis by mass spectrometry. Indeed, twelve VP2 peptides were detected (Fig. S4), verifying the biosynthesis of the correct VP2 protein. Remarkably, when comparing the relative VP2 content in constant amounts of TSP between different transformants, the intensity of the cross-reacting band differed significantly among individual transformants (Fig. 3a), fully consistent with the flow cytometry data. For the LS-lacking construct, VP2-His_6_ was only weakly detectable in eight out of 15 transformants at the given exposure time, while two transformants (T5 and T11) had very high levels under the same conditions (Fig. 3a). Similarly, for the LS-containing construct, one transformant (T3) showed the maximum and very high VP2 levels (Fig. 3b), while the protein was only weakly detectable (at a level of approx. 5% of T3) in most other transformants (Fig. 3). The high VP2 levels in a few, very specific transformants further supported the idea that the integration of multiple copies of expression cassettes and/or yet unknown positional effects strongly enhanced transgene expression from the EF promoter.

### VP2 yield analyses in the best *N. oceanica* transformants

**Fig. 4:**
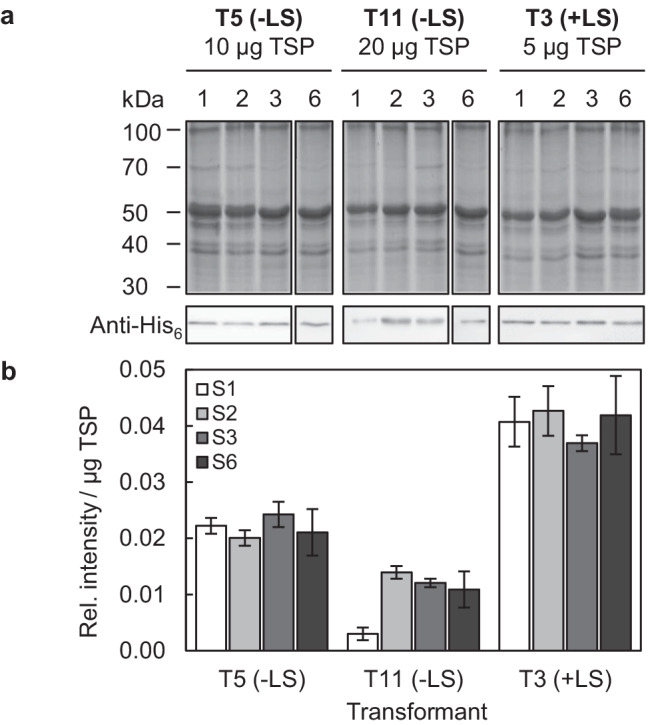
Analysis of the effect of the subculture number on VP2 production in the best transformants. **a** VP2-His_6_ was quantified in extracts of TSP isolated from batch cultures that had been harvested at mid-exponential growth phase (OD 1.0–1.3) and subjected to different numbers of subcultures (Fig. S5). To obtain signals of cross-reactivity of similar intensity and within the relatively narrow dynamic range of quantification, different amounts of TSP were loaded for the three transformants producing VP2 at moderate (T11, 20 µg TSP), high (T5, 10 µg), and maximum levels (T3, 5 µg). **b** The relative VP2 content was calculated based on the relative intensity of VP2-His_6_ cross-reactivity by dividing the absolute intensity by that of a His_6_-tagged loading control and normalizing this ratio to µg TSP. For each subculture, the mean of the relative VP2 content (± SD) of two technical replicates of immunoblotting is given

For biotechnological vaccine production at a large scale, recombinant protein productivity shall be robust, i.e., constant over a wide growth phase and rather independent of transformant generation and culture age. To investigate the robustness of VP2 production, the best three transformants (T3, T5, and T11) were subjected to long-term analysis of VP2 production over many generations in multiple subcultures. Individual transformants were grown in 100-ml batch cultures from a starting OD_540_ = 0.05 to mid-log phase (OD_540_ 1.0–1.3), diluted in fresh f/2 medium, and regrown several times (Fig. 4). The batch cultures had a mean growth rate of µ = 0.37 d^−1^ (Fig. S5), and each subculture corresponded to four to six generations. Cells of different subculture numbers were harvested, and the relative VP2 content was compared between samples and transformants based on the relative signal intensity of the cross-reactive 50-kDa band normalized to µg TSP. To obtain similar intensities of cross-reactivity that were located within the relatively narrow dynamic range of detection, the amounts of loaded TSP were varied (5–20 µg) and adjusted to the VP2 level of each transformant (Fig. 4). For different amounts of TSP of the same transformant, the calculated relative VP2-His_6_ content was constant (data not shown). The VP2 content remained rather constant and did not detectably decrease with increasing number of subcultures (Fig. 4). For T11, which showed the lowest VP2 content of the three transformants, some variations were measured between subcultures (Fig. 4b). For the two transformants with the highest VP2 content (T3 and T5), however, the values remained almost stable over all subcultures analyzed (Fig. 4b). Thus, the VP2 content was rather constant, particularly in the two best overproduction strains, and was independent of the transformants’ age and the number of subcultures, indicating robust VP2 production.

**Fig. 5:**
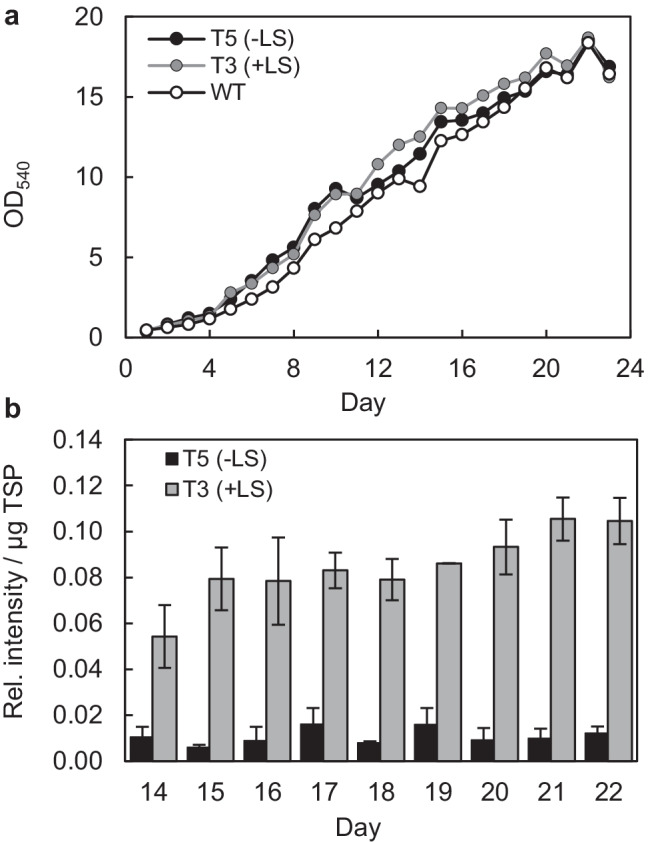
Growth analyses and relative VP2 content of two *N. oceanica* transformants cultivated in PBRs. **a** Growth curves of the most productive two transformants are shown in comparison to the wild type (WT). **b** The VP2 content (in relative intensity of anti-His_6_ cross-reactivity per µg TSP) was estimated between days 14 and 22. Mean values and SD of two technical replicates of immunoblotting are shown for one (PBR1) of two biological replicates (Fig. S7). VP2 yields were calculated for day 20 (see Table 1) by a calibration curve of pure VP2-His_6_ and by quantitative anti-VP2 and anti-His_6_ immunoblotting (Figs. S8–10)

Furthermore, it is desirable that recombinant protein production continues at a high rate until the early stationary phase and that protein integrity is preserved at high cell densities, for instance, without quality reduction by proteolytic degradation. To address this concern, the best two transformants (T3 and T5) were selected for cultivation in small-scale PBRs to perform *in-depth* kinetic analyses of VP2 yield, productivity, and integrity. A constant supply of CO_2_/air (1%/99% (v/v)) during the light period and fivefold elevated concentrations of nitrate and phosphate enabled microalgae proliferation up to 4.0*10^9^ cells/ml (approx. OD_540_ = 20; Fig. 5a). After an initial exponential growth phase, during which the cultures typically grew to an OD_540_ of 9 within 9–10 days, the algae entered a more linear growth phase until reaching the early stationary phase after day 20. Notably, the growth characteristics of both *VP2* expressing transformants were very similar to those of the wild-type strain in terms of growth rates and growth phase length, indicating that VP2 biosynthesis and the presence of hygromycin had, if at all, only a minor negative impact on these parameters (Fig. 5a). Only in rare cases was the growth rate of a transformant culture noticeably lower and resulted in a lower maximum OD_540_ (data not shown).

To investigate whether the ultrastructure of *N. oceanica* was altered upon *VP2* expression, the cells were analyzed by transmission electron microscopy. Cells cultivated in PBRs were harvested in the mid-exponential growth phase, and the membrane lipids were stained with osmium tetroxide. In addition to the cell nucleus, mitochondria, and peroxisomes, *N. oceanica* cells generally contained one large chloroplast with long thylakoid membranes stacked in very few layers over nearly the entire organelle length (Fig. S6). Additionally, one or a few small vacuoles (0.3–0.5 µm in diameter) generally appeared “empty” in the wild type. All these organelles are typical for *Nannochloropsis*, if cultured under standard growth conditions (Fig. S5a, Roncaglia et al. 2021). In both transformants, however, the small vacuoles often contained short, regular, and tightly packed tubules that were stained intensively dark (Fig. S6b, c). These tubule-containing vacuoles were slightly more pronounced in T3 (with higher VP2 yield) than in T5 (Fig. S6b, c), indicating intracellular remodeling upon *VP2* expression. The vacuolar content might represent thylakoid membranes that are being degraded as part of constitutive autophagy of chloroplasts, referred to as chlorophagy (Izumi et al. 2017).

PBR cultivation allowed sufficient biomass production for kinetic analyses of the relative VP2-His_6_ content (Fig. 5a, b). Despite minor variations, the relative VP2 content of both transformants remained relatively stable over the entire growth period analyzed (days 14 to 22, Fig. 5b). Importantly, no protein degradation was detectable over the entire growth period according to anti-His_6_ immunoblotting analyses (Fig. S7, data not shown). To calculate the absolute VP2 content, VP2-His_6_ was purified from one *N. oceanica* transformant (T3, + LS, day 23 of PBR1). Because the C-terminus is not surface-exposed in subviral particles formed by 20 recombinant VP2 trimers (Coulibaly et al. 2010), the protein was purified by Ni–NTA under denaturing conditions in 8 M urea to high homogeneity of 86% (Fig. S8a). The concentration of pure VP2 was determined by SDS–PAGE and by relating the silver-stained band intensity of VP2-His_6_ to that of a BSA calibration curve (25–150 ng, Fig. S8b). A standard curve of purified VP2-His_6_ detected by anti-VP2 antibodies allowed absolute quantification of VP2-His_6_ in TSP fractions by immunoblotting (Table 1, Fig. S9). The same VP2 content of both transformants (< 0.2% difference) was determined if the VP2 calibration curve was analyzed by anti-His_6_-specific immunoblotting (Fig. S10).

For T5 lacking the LS, 1.4% VP2 of TSP was determined at the end of the linear growth phase (day 20), as representatively shown for one PBR culture (PBR1, Fig. 5a) of two biological replicates (Table 1). For T3, the VP2 level was even higher, yielding 4.4% of TSP (Table 1). The VP2 yield per culture volume if harvested after 20 days was approx. 2.6 mg VP2*l^−1^ (for T5) and 5.7 mg*l^−1^ (T3, Table 1). Determination of the BDW of the PBR cultures on day 20 allowed VP2 yield normalization and the calculation of 1.5 mg VP2 per g of BDW for T5 and 3.1 mg*g^−1^ BDW for T3 (Table 1).

### Analysis of transgene copies

**Fig. 6:**
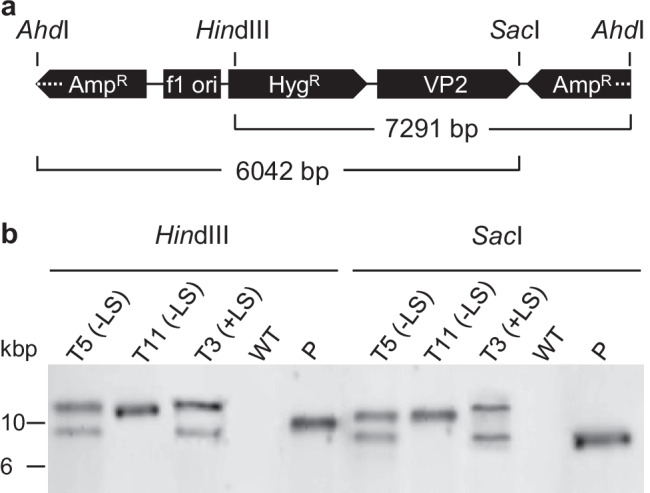
Determination of the insertion number of the expression vector in selected *N. oceanica* transformants by Southern blotting. **a** The schematic diagram gives the expected minimum fragment sizes of the *Ahd*I-linearized expression vector upon integration into the nuclear genome of the transformants and after DNA digestion with *Hin*dIII or *Sac*I. **b** Total DNA of the three transformants was digested with one enzyme and hybridized with a *Hyg*^*R*^-specific probe. Digested wild-type DNA (WT) served as a negative control. The digested plasmid (P, i.e., pNoc ox Pro(EF)::VP2) served as a positive control for successful probe hybridization (approx. 9 kbp). Outside of the selected blot region, labeled DNA fragments were not detected. One single insertion was detected for T11 and at least two insertions for T5 and T3, reproducibly for both restriction enzymes

To address whether the high VP2 productivity in three specific transformants was caused by multiple insertions of the expression vector into the nuclear genome, we analyzed the insertion number by Southern blotting. Total DNA was isolated, digested with either *Hin*dIII or *Sac*I, and hybridized with a labeled *Hyg*^*R*^-specific DNA probe to detect the expression cassette. The absence of any detectable band for the wild type confirmed the probe specificity (Fig. 6). Because the plasmid had been linearized with *Ahd*I prior to *N. oceanica* transformation and contained single sites for each of *Hin*dIII and *Sac*I, the DNA fragments containing an integrated expression cassette had expected sizes of ≥ 7.3 kbp (for *Hin*dIII) and ≥ 6.0 kbp (for *Sac*I, Fig. 6a). This was indeed the case (Fig. 6b). The same number of expression plasmids integrated into the nuclear genome was determined consistently for both restriction endonucleases. While a single expression cassette was detected for T11, each of the two most productive transformants (T3 and T5) contained at least two copies of the expression vector. To further confirm the number of expression cassettes, a *VP2*-specific probe was used, and the same numbers and sizes of labeled DNA fragments were detected (data not shown). Together, the results strongly suggested a direct causal relationship between the increased number of integrated expression cassettes and the high level of transgene expression in the same transformants.

## Discussion

The main target for vaccine development against IPNV infection of fish is the capsid VP2 protein because of its major antigenic epitopes and cell attachment function. These features make it an ideal model vaccine for recombinant protein production in *N. oceanica* upon nuclear transformation. Among several *N. oceanica* promoters previously tested, the EF and NR promoters yielded maximum *Venus* expression and protein yields (de Grahl et al. 2020) and were chosen here for *VP2* expression. As the expression rates of transgenes from the same promoter often differ significantly due to positional effects (Schroda 2019), we first generated VP2-Venus fusions (Fig. 1a) and analyzed transformant populations to be able to compare different genetic element combinations in an efficient manner (Ramarajan et al. 2019; Schroda 2019). The strategy of averaging Venus fluorescence in transformant populations proved suitable to generate reproducible results on both gene expression by qRT–PCR and recombinant protein production by flow cytometry (Figs. 1a and 2; de Grahl et al. 2020).

Both the EF and NR promoters showed a comparable strength in *Venus* expression in our previous study (de Grahl et al. 2020), but similar analyses for the VP2-Venus transformant populations by qRT–PCR carried out here revealed pronounced differences, namely, a > 13-fold lower expression of the *VP2-Venus* gene from the NR promoter compared with the EF promoter (Fig. 1a). These results were consistent with the median Venus fluorescence values determined by flow cytometry because hardly any Venus fluorescence was detectable in the NR promoter populations (Fig. 2a, Fig. S2). The strength differences between the two promoters were most pronounced when only the 10% of cells with the highest Venus fluorescence were averaged; the median fluorescence was sixfold higher for the EF promoter population than for the corresponding NR promoter (Fig. 2b). This 10% subpopulation is most relevant for the generation of high-yield expression constructs and strains and eventually allows the identification of individual reporter-free transformants with the highest protein yield by cell sorting (Fig. 3).

The low *VP2* expression from the NR promoter was unexpected and contrasted with the high strength of the LS-containing NR promoter when driving *Venus* expression alone without any gene fusion in our previous study (de Grahl et al. 2020). A similar variability in transgene expression from the same promoter has been observed in *C. reinhardtii* upon nuclear transformation (Schroda 2019). The underlying molecular mechanism is most likely related to the fact that the insertion of different transgenes into the nuclear genome can affect histone binding and nucleosome formation at the promoter-transgene junction (Chereji and Clark 2018). For specific “incompatible” promoter-transgene combinations, this leads to an occlusion of *cis*-regulatory elements and a reduction in the binding of *trans*-activators and transcription factors, strongly reducing gene expression. In addition, the typically low degree of histone acetylation in transgenes, as often observed in *C. reinhardtii*, promotes histone binding to *cis*-regulatory elements and further hinders promoter accessibility (Fu et al. 2015). Such occluded *cis*-regulatory elements can only be accessed by strong *trans*-activators (Cairns 2009). Other promoters, however, possess a rather high general affinity for histones and stimulate nucleosome packing. The strength of these promoters remains largely unaffected by different downstream transgenes. The same holds true for promoters regulated by strong activators. In contrast, the accessibility of promoters with low histone affinity, unfavorable nucleosome packaging, and/or regulation by weak activators is strongly dependent on the transgene sequence. This interdependence is typical for conditional/inducible promoters (Cairns 2009) and may explain the observed differential strength of the nitrate-inducible NR promoter in *Venus* and *VP2-Venus* expression (de Grahl et al. 2020).

Interestingly, the extension of the EF promoter by its own LS of only 14 amino acid residues indeed enhanced *VP2-Venus* expression by approx. 40% (Fig. 1). This effect continued to the protein level, because the median Venus fluorescence of the highest 10% subpopulation was increased by 60% (Fig. 2b). We previously observed the same stimulatory effect of the LS of the NR promoter on *Venus* expression (de Grahl et al. 2020). Similar LS enhancer effects have been reported for *E. coli* (Sprengart et al. 1996), *Synechocystis* (Betterle and Melis 2018), and the chloroplasts of *C. reinhardtii* (Richter et al. 2018) and tobacco (Kuroda and Maliga 2001; Ye et al. 2001). By fusing a native gene that is highly expressed in cyanobacteria to the gene of interest (e.g., the ß-subunit of phycocyanin), different proteins of eukaryotic plant and animal origin were stabilized by this extended leader sequence in Synechocystis sp. PCC 6803, and a high accumulation of 10–20% of the total cellular protein could be achieved (Betterle et al. 2020; Zhang et al. 2021). As a downside, this promising effect is often specific for certain combinations between gene elements (promoter, LS, and transgene). Thus, the effects are largely unpredictable and require additional combinatorial steps of expression cassette optimization (Richter et al. 2018). For *N. oceanica*, the expression-enhancing effect of one specific LS in combination with different promoters and transgenes likewise turned out to be unpredictable because *Venus* expression driven by the EF promoter was only marginally enhanced by the presence of the same LS in our previous study (de Grahl et al. 2020). Mechanistically, an LS may either stabilize the mRNA or enhance transgene translation (Richter et al. 2018). Alternatively, an LS may also alter histone binding, nucleosome formation, and promoter accessibility and activity, as discussed above for the transgene effect on promoter strength. In future studies, the expression-enhancing effect of EF-LS observed in *N. oceanica* can be used to boost transgene levels but all combinations of expression cassette elements need to be carefully re-evaluated. Promising future improvement potential also lies in the application of chimeric promoter-LS constructs, i.e., combining, for instance, the strong EF promoter with the LS of NR.

The biological activity of recombinant proteins should not be compromised by partial misfolding or sterical hindrance by bulky reporters or tags. We therefore omitted the fluorophore in the subsequent analyses (Fig. 1b). The identical N-terminal location of VP2 in both constructs was predicted to maintain protein production at equally high levels from the same promoter. Indeed, upon expression from the EF promoter, reporter-free *VP2* reached an even higher transcript level than the reporter-tagged construct, as deduced from qRT–PCR analysis of transformant populations (Fig. 1a, b). The C-terminal His_6_-tag of VP2 allowed analyses of VP2 content in individual transformants by immunoblotting. Among the 15 analyzed transformants for each EF promoter construct (± LS), high variations in the VP2 level were detectable (Fig. 3). Most transformants (16 out of 30) showed a very low level of VP2-His_6_, which corresponded to approximately 5–10% of the levels of the best producer strains, and are interpreted as a basal VP2 level without major positional effects in enhancing transgene expression. Two LS-lacking transformants (T5 and T11) and one LS-containing transformant (T3), however, showed strongly elevated VP2 levels and were most promising for VP2 production. Southern blot analyses demonstrated that those two transformants with the highest VP2 yield (T3 and T5) possessed two copies of the full-length expression vector integrated into the genome (Fig. 6b), while transformant T11 with moderate VP2 yield (Fig. 4) had a single copy. Thus, the double insertion of the VP2 expression cassettes is most likely an important factor for boosting VP2 yields in T3 and T5. For *C. reinhardtii*, a very similar correlation between the number of transgene insertions and the expression level of a reporter gene was reported recently (Shahar et al. 2019). Furthermore, because the VP2 level of T11 with its single cassette copy was much higher than that of the other 27 screened transformants (Fig. 3), pronounced positional effects due to random genome integration are most likely responsible for its elevated transcriptional activity at the given integration site.

An important prerequisite for the cultivation of transgenic microalgae at the industrial scale is strain stability in terms of constant recombinant protein production over generations. This is particularly important for *Nannochloropsis*, as long-term cryo-conservation is insufficiently established, and strain maintenance requires regular, time-consuming subculturing under low light and temperature. When analyzing the VP2 level and stability over approximately 30 generations, the yield was nearly constant and did not detectably decline with increasing generation number (Fig. 4), making the expression system suitable for cultivation in large PBRs.

Microalgal productivity, i.e., the daily rate of recombinant protein production, is generally the highest between the end of the linear (or exponential) phase and early stationary growth phase. In this productive phase, however, several factors may reduce protein yield and quality, including nutrient limitation, insufficient light penetration, increased proteolytic activity, or the induction of autophagy. To determine the optimal time point of cell harvest, we analyzed the kinetics of VP2 production in PBR cultures in the linear growth phase until the early stationary phase (Fig. 5a). For the best LS-lacking transformant (T5), the VP2 yield showed minor variations over the growth period and a moderate yield of 1.4% VP2 in TSP at the end of the linear growth phase (day 20, Table 1). The best transformant T3 included the LS and produced VP2 at a high level of 4.4% VP2 of TSP (3.1-fold higher than T5), which was equal to a yield of 5.7 mg*l^−1^ VP2 and 3.1 mg*g^−1^ BDW after 20 days of cultivation (Table 1). Importantly, no VP2 degradation was detectable by immunoblotting, not even in the early stationary growth phase. Importantly, protein production did not negatively impact the growth rate of *N. oceanica* compared to the wild-type strain (Fig. 5a), which demonstrates the high robustness of this *Nannochloropsis* expression system.

In the transformants capable of high VP2 production, transmission electron microscopy revealed interesting atypical ultrastructures in which many vacuoles contained stacked tubules (Fig. S5b,c), which were only rarely seen in the wild type. Such structures have been previously described in *N. oceanica* and other Eustigmatophytes, where they specifically occurred upon nitrogen starvation. Most likely, these tubules originate from thylakoid degradation as a consequence of chlorophagy, followed by vacuolar degradation of photosynthetic protein complexes to amino acids (Roncaglia et al. 2021). Typically, this process is paralleled by a reduction in algal protein content. The presence of similar structures in *VP2*-expressing cells indicates a partial cellular remodeling by constitutive or (VP2-) inducible chlorophagy (Heredia-Martínez et al. 2018) to refill amino acid pools for VP2 biosynthesis.

Good yields of recombinant proteins upon nuclear transformation of microalgae have been reported for Venus in *N. oceanica* (4.9% of TSP, de Grahl et al. 2020), followed by two proteins of biotechnological relevance, namely, an antimicrobial peptide in *N. oculata* (4.3% of TSP, Li and Tsai 2009) and a human monoclonal antibody in *Phaeodactylum tricornutum* (8.7% of TSP, Hempel et al. 2011). In contrast, the protein levels generally remained minimal in *C. reinhardtii*, particularly upon nuclear expression (often < 1% of TSP, Rosales-Mendoza et al. 2012; Specht and Mayfield 2014). The promising yields achieved upon nuclear transformation in *N. oceanica* in this study (4.4% VP2 of TSP) may shift the former focus from *Chlamydomonas* (Dobos and Roberts 1983; Fu et al. 2015) to this newly emerging production alga. Novel molecular genetic tools and methods that have been developed in the past few years (de Grahl et al. 2020; Poliner et al. 2020, 2018a) will help to further develop and optimize *N. oceanica* as an expression host.

In summary, it was indeed possible to produce the viral surface protein VP2 of IPN virus as the first model subunit vaccine in *N. oceanica* and to achieve a high yield of 4.4% of TSP. Recombinant protein production was rather constant over many generations and robust with respect to proteolytic stability and protein integrity and its independence of the growth phase. Despite minor apparent remodeling of cell organelles upon *VP2* expression, the transformants’ physiology in terms of growth rate and maximum OD remained comparable to that of the wild-type strain. The most productive transformants contained two insertions of the expression cassette in the nuclear genome, and transgene expression was most likely further boosted due to positional enhancer effects caused by the genomic environment of very specific genomic integration sites. Identification of these genomic integration sites and targeted integration of other transgenes into the same site by CRISPR/Cas gene editing technology might further improve the production of recombinant proteins in *N. oceanica*.

In future studies, the VP2-containing biomass of these transformants should be added to fish feed for oral fish vaccination and analysis of its immunogenic protection capability against IPNV infections. When the VP2 protein was produced in *Saccharomyces cerevisiae*, a specific immune response in rainbow trouts was indeed induced when feeding lyophilized yeast cells (Allnutt et al. 2007). Because *Nannochloropsis* has a very thick and rigid cell wall (approximately 0.1 µm), which is made of an inner cellulose and an outer hydrophobic algaenan layer (Scholz et al. 2014), the alga can probably even better serve for bioencapsulation of the VP2 protein compared to baker’s yeast. Bioencapsulation of a vaccine in *Nannochloropsis* improves antigen protection against partial denaturation and cleavage in the acidic environment of the stomach and enhances immune responses after protein release in the fish intestine (Charoonnart et al. 2018). The application strategy of feeding entire microalgae also circumvents the need to extract high-value products, which is challenging due to the rigid cell wall and small size of *Nannochloropsis*. The expression cassette of the hygromycin resistance gene can be eliminated by CRISPR/Cas9 technology established for *N. oceanica* (Poliner et al. 2018b; Naduthodi et al. 2019). The first successful production of a viral surface protein in *N. oceanica* reported here allows the relatively straightforward transfer of strategies and methodologies to other subunit vaccines for diverse applications in aquaculture and agriculture, e.g., the closely related VP2 protein of IBDV, which infects poultry and causes high mortality rates (Sharma et al. 2000).

## Supplementary Information

Below is the link to the electronic supplementary material.

Supplementary file1 (PDF 1528 KB)

Supplementary file2 (PDF 124 KB)

## Data Availability

The authors will make scientific material available upon request.
