# Supplementary material for: Production of a viral surface protein in Nannochloropsis oceanica for fish vaccination against infectious pancreatic necrosis virus
Source: Appl Microbiol Biotechnol. 2022 Sep 7;106(19-20):6535–49. doi: 10.1007/s00253-022-12106-7 (PMC9449291; doi:10.1007/s00253-022-12106-7)
Supplement: Supplementary file 1 — Supplementary file1 (PDF 1528 KB) [file 253_2022_12106_MOESM1_ESM.pdf]

"Production of a Viral Surface Protein in *Nannochloropsis oceanica* for Fish Vaccination against Infectious Pancreatic Necrosis Virus"

Journal: *Applied Microbiology and Biotechnology*;

Authors: Sweta Suman Rout, Imke de Grahl, Xiaohong Yu, and Sigrun Reumann;
Affiliation: Plant Biochemistry and Infection Biology, Institute of Plant Science and Microbiology, University of Hamburg, D-22609 Hamburg, Germany;
Email address of corresponding author: sigrun.reumann@uni-hamburg.de

SUPPLEMENTARY MATERIAL

Figure S1

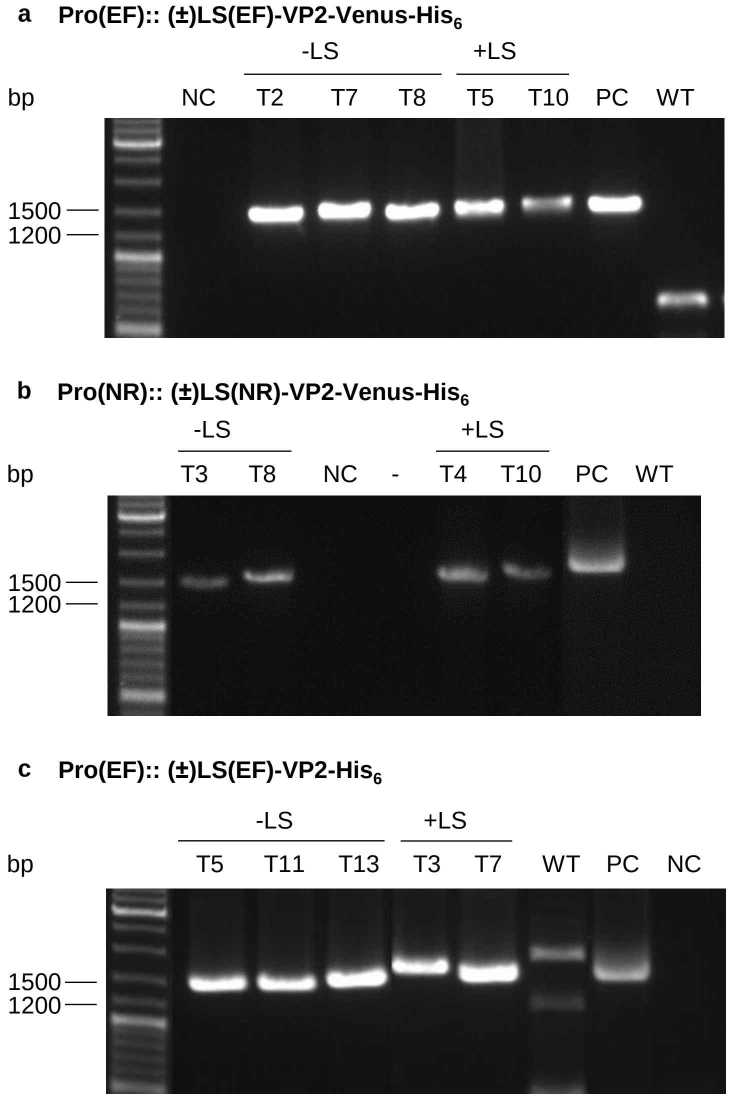

Figure S1 Verification of the expression vector presence in *N. oceanica* in individual transformants by PCR. Genomic DNA was isolated from few transformants of *N. oceanica*. The presence of the expression construct was verified with two primers amplifying parts of the expression cassettes (Table S2). **a** For VP2-*Venus-His*$_6$ expressed from the elongation factor (EF) promoter, the expected band sizes were 1509 bp (-LS) and 1551 bp (+LS), and 1509 bp for the positive control (PC; pNoc ox Pro(EF)::VP2-Venus). No amplicons of the same sizes were detected for the wild type (WT) and the negative control (NC). The NC lacked any DNA. **b** For the *VP2-Venus* constructs expressed from the NR promoter (±LS), the expected band sizes were 1515 and 1558 bp, respectively. For the PC, pNoc ox Pro(NR)::LS(NR)-VP2-Venus plasmid DNA, an amplicon of 1558 bp was obtained. No amplicons were detected for the wild type (WT). **c** For the reporter-free EF::VP2 constructs (±LS), the expected band sizes were 1572 bp and 1614 bp, respectively. The plasmid pNoc ox Pro(EF)::LS(EF)-VP2-His$_6$ was used as a PC (expected size 1614 bp) and no amplicons of the same size were detected for the wild type (WT)

Figure S2

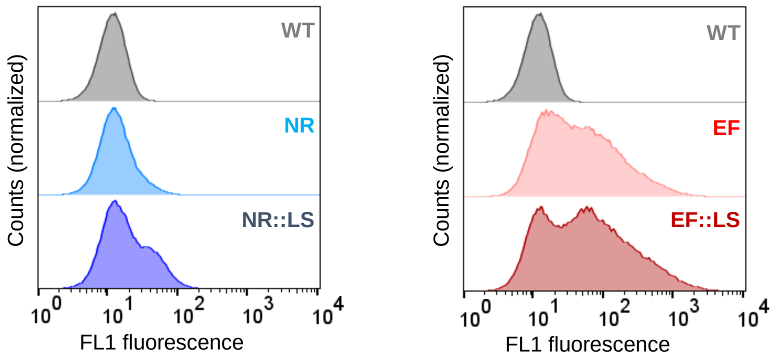

Figure S2 Histograms of FL1 fluorescence in four different populations of *N. oceanica* transformants analyzed by flow cytometry. FL1 fluorescence distribution in the wild-type (WT, gray) and VP2-Venus populations using either the NR or EF promoter (± LS). The wild type (gray) showed very low autofluorescence compared to Venus fluorescence of the transformant populations

Figure S3

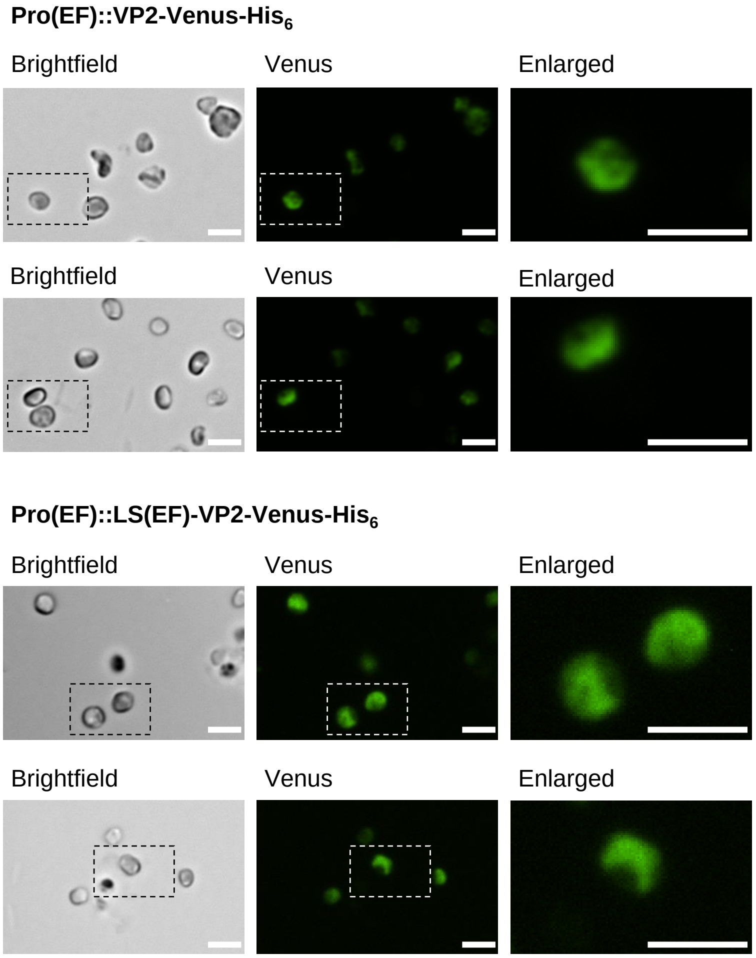

Figure S3 Confocal microscopy of *N. oceanica* EF promoter transformants. Representative images of two stable *N. oceanica* transformants producing VP2-Venus-His$_6$ showed strong cytosolic Venus fluorescence for both EF promoter versions with and without LS. Scale bar: 5 µm. Dashed boxes show enlarged regions

Figure S4

MNTNKATATYLKSIMLPETGPASIPDDITERHILKQETSSYNLEVSESGSGILVCFPGAPGSRIGAHYRWNANQTGLEFD
QWLETSQDLKKAFNYGRLISRKYDIQSSTLPAGLYALNGTLNAATFEGSLSEVESLTYNSLMSLTTNPQDKVNNQLVTKG
VTVLNLPTGFDKPYVRLEDETPQGLQSMNGAKMRCTAAIAPRRYEIDLPSQRLPPVTATGALTTLYEGNADIVNSTTVTG
DINFSLTEQPAVETKFDFQLDFMGLDNDVPVVTVVSSVLATNDNYRGVSAKMTQSIPTENITKPITRVKLSYKINQQTAI
GNVATLGTMGPASVSFSSGNGNVPGVLRPITLVAYEKMTPLSILTVAGVSNYELIPNPELLKNMVTRYGKYDPEGLNYAK
MILSHREELDIRTVWRTEEYKERTRVFNEITDFSSDLPTSKAGSGHHHHHH

Figure S4 Amino acid sequence of VP2-His$_6$ with marked peptides identified by mass spectrometry. A band of approximately 50 kDa of the LS-lacking transformant T5 was cut out of the SDS-PAGE gel and subjected to a tryptic in-gel digest. After chromatographic peptide separation, the eluted peptides were analyzed on a Quadrupole Orbitrap mass spectrometer, and the obtained LC–MS/MS data were searched against appropriate databases (see Material and Methods for details). In total, twelve VP2-specific peptides (marked in red) were identified, which covered 48% of the total VP2-His$_6$

Figure S5

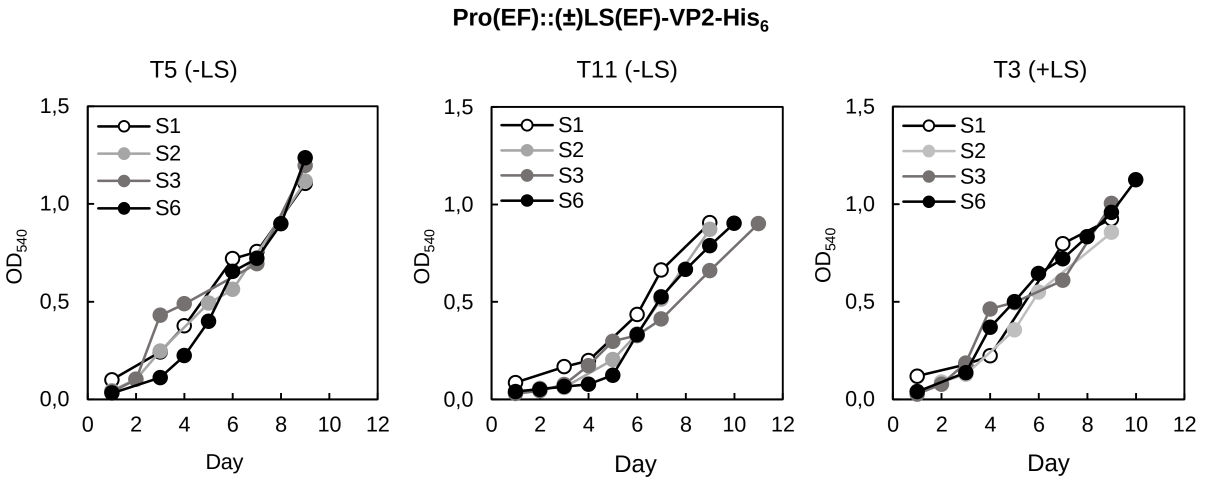

Figure S5 Growth curves of *VP2*-expressing transformants in batch cultures subjected to different numbers of subcultures. Upon reaching an OD_{540} of 1.0-1.3, each transformant culture was transferred six times to 100 ml fresh f/2 medium, equal in total to approx. 25 generations. Prior to another subculture (S), the majority of cells were harvested for VP2 yield analyses by anti-His_6 immunoblotting (Fig. 4)

Figure S6

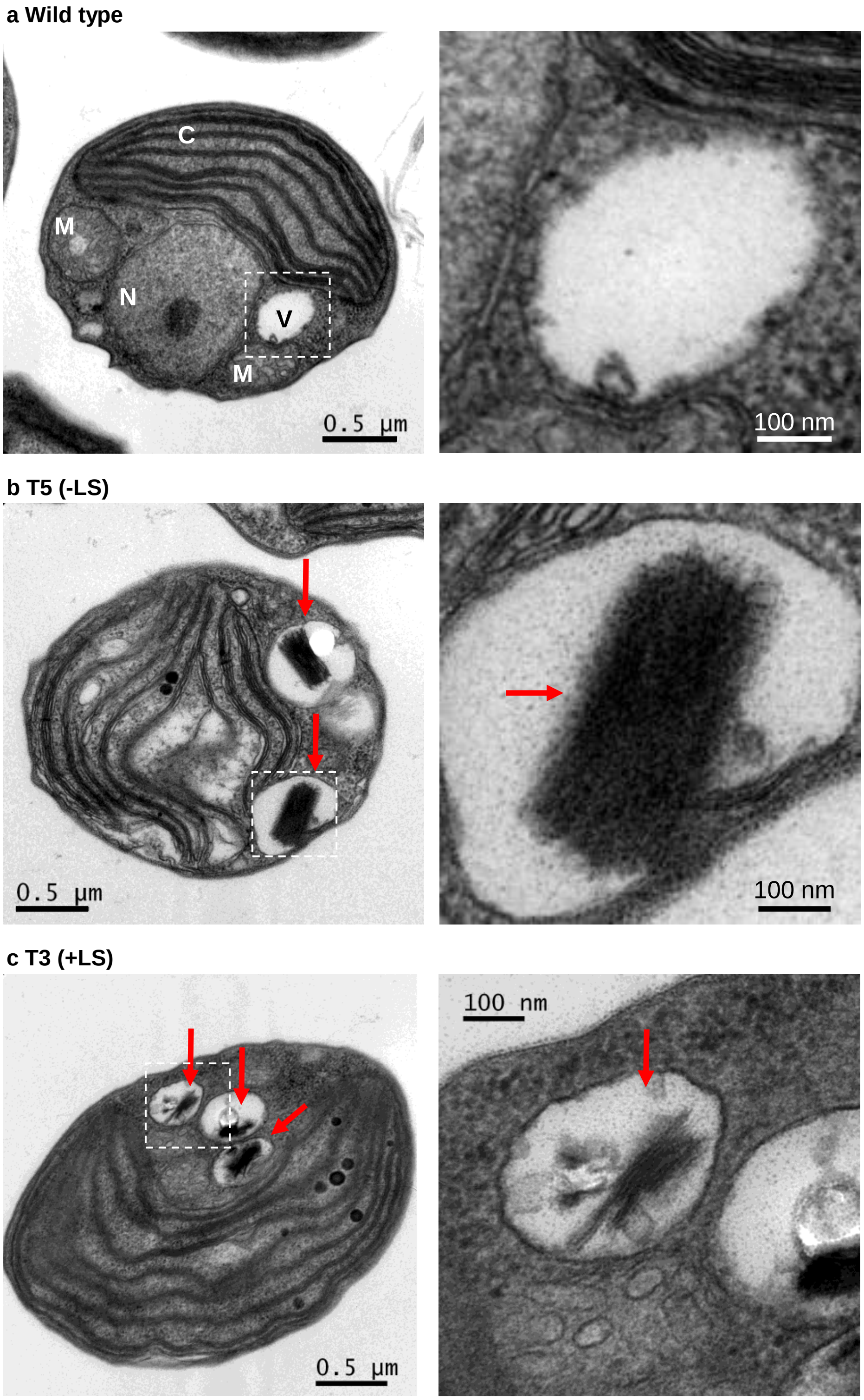

Figure S6 TEM images of the wild type and two VP2 overproducing transformants. The cells were cultivated in PBRs to mid-exponential growth phase, harvested, fixed and stained with osmium tetroxide. **a** Representative wild-type cells from nonstressed growth conditions contain one chloroplast (C), the nucleus (N), few mitochondria (M) and one empty vacuole (V). **b** and **c** The vacuoles of transformants T5 and T3 often contained densely packed, stacked tubular-like structures (red arrows) that may represent thylakoid membranes originating from chlorophagy. Scale bars as indicated. Dashed squares show enlarged regions

Figure S7

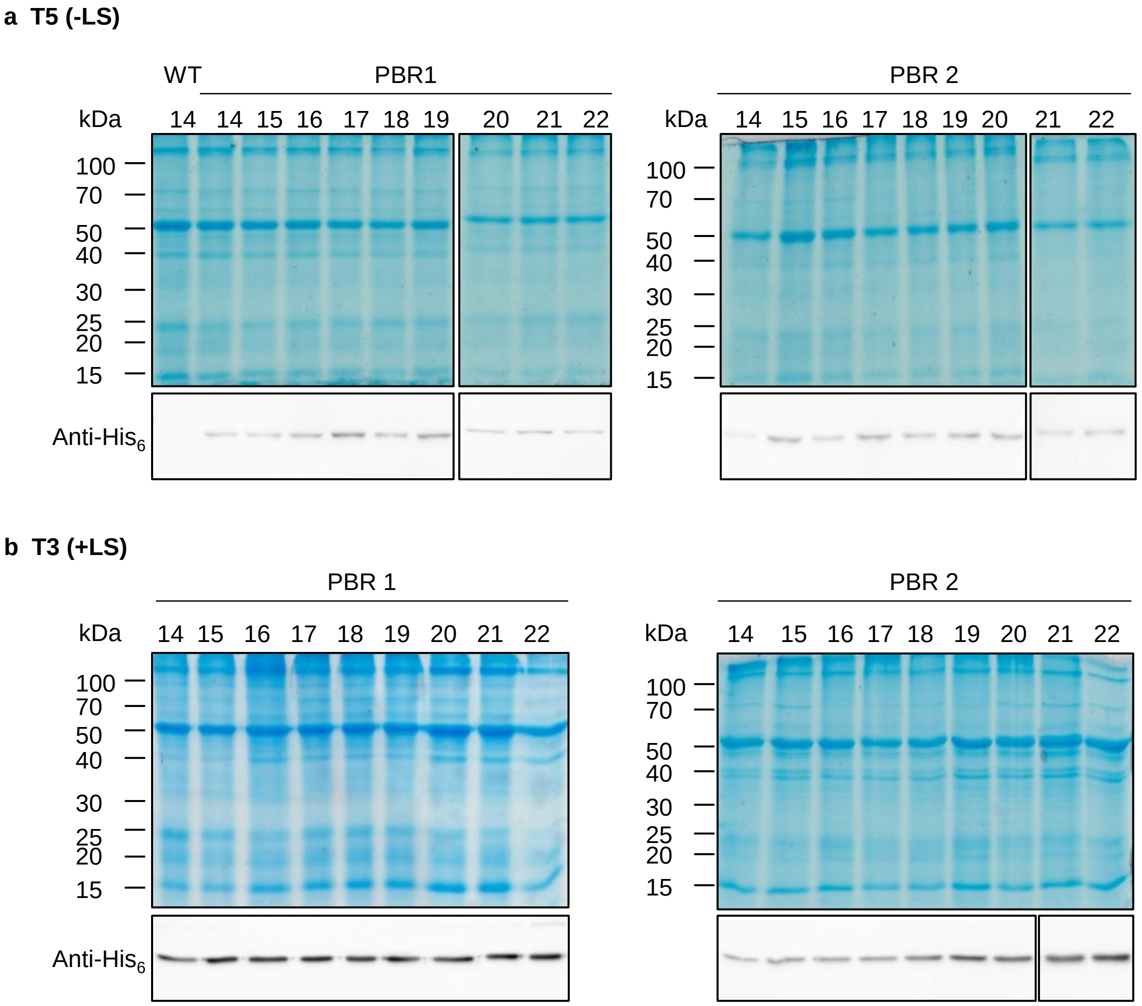

Figure S7 Kinetic analyses of VP2-His$_6$ content in *N. oceanica* transformants during cultivation. a For transformant T5 (-LS), 10 µg of TSP were loaded for both immunoblotting and Coomassie staining. **b** For transformant T3 (+LS) 5 µg of TSP (immunoblotting) and 10 µg (Coomassie gel) were used. The VP2 protein yield was quantified from Days 14 to 22 using two biological replicates (PBR1 and 2). Analysis of the VP2 content in cellular extracts of TSP was carried out by immunoblotting with an anti-His$_6$ specific antibody and a purified His$_6$-tagged standard protein (14 kDa) as a loading control. The antibody did not cross-react with any soluble 50-kDa protein of the wild type (WT). The Coomassie (CBB) stained SDS-PAGE gel served as a loading control. The relative signal intensity of cross-reactivity normalized to µg TSP of two technical replicates is shown in Fig. 5

Figure S8

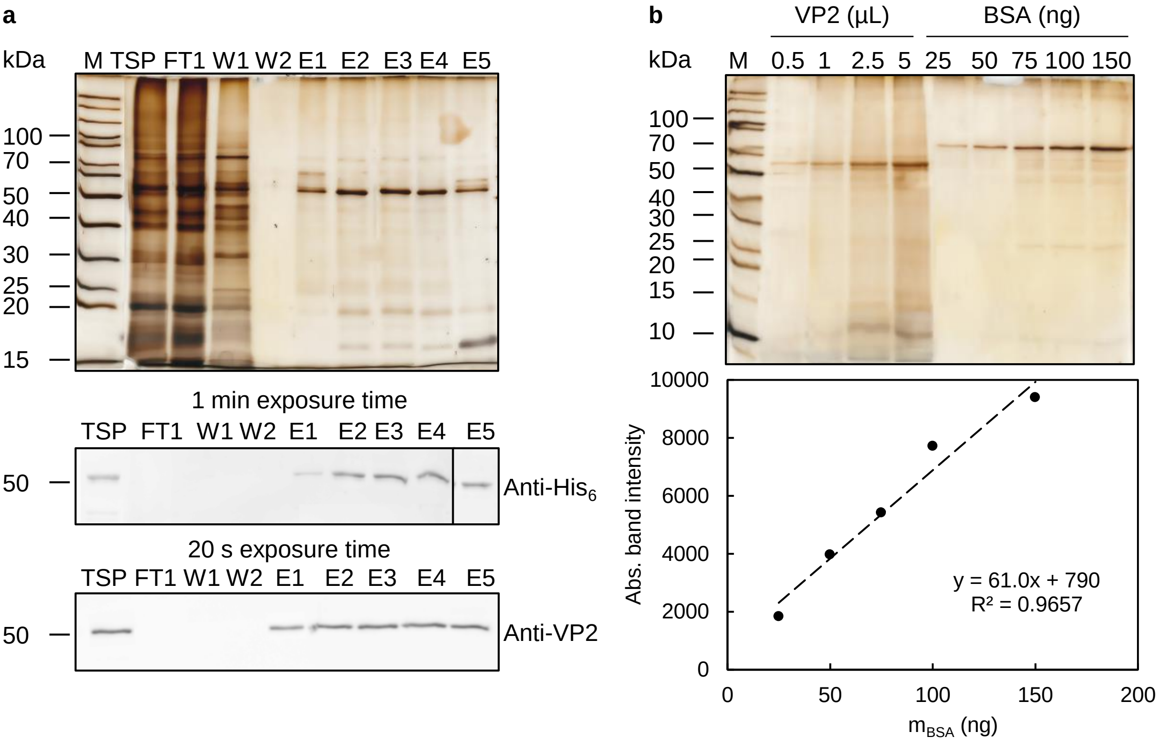

Figure S8 VP2 purification under denaturing conditions by Ni-NTA affinity chromatography and quantification by SDS-PAGE and silver staining. a VP2-His$_6$ was purified from one transformant (T3, +LS, Day 23 of PBR1) under denaturing conditions in 8 M urea by Ni-NTA. The fractions were analyzed by using constant volumes (TSP, FT, and W: 4 µl; E: 1 µl) by SDS-PAGE, followed either by silver staining or immunoblotting. The identity of the 51-kDa band with VP2 was verified by immunoblotting with an anti-His$_6$ or an anti-VP2 antibody. The purity of VP2 was quantified by ImageJ (86% for E2-E4; 73% for E1 and E5). **b** The eluates E2-E4 were subsequently pooled, and the concentration of pure VP2-His$_6$ was determined by loading different volumes and calculating the amount of pure VP2 (for 5 µl) based on its band intensity and a BSA calibration curve (25-150 ng BSA), yielding 18 ng/µl. Abbreviations: E, eluate; FT, flow-through fraction; W, wash fraction

Figure S9

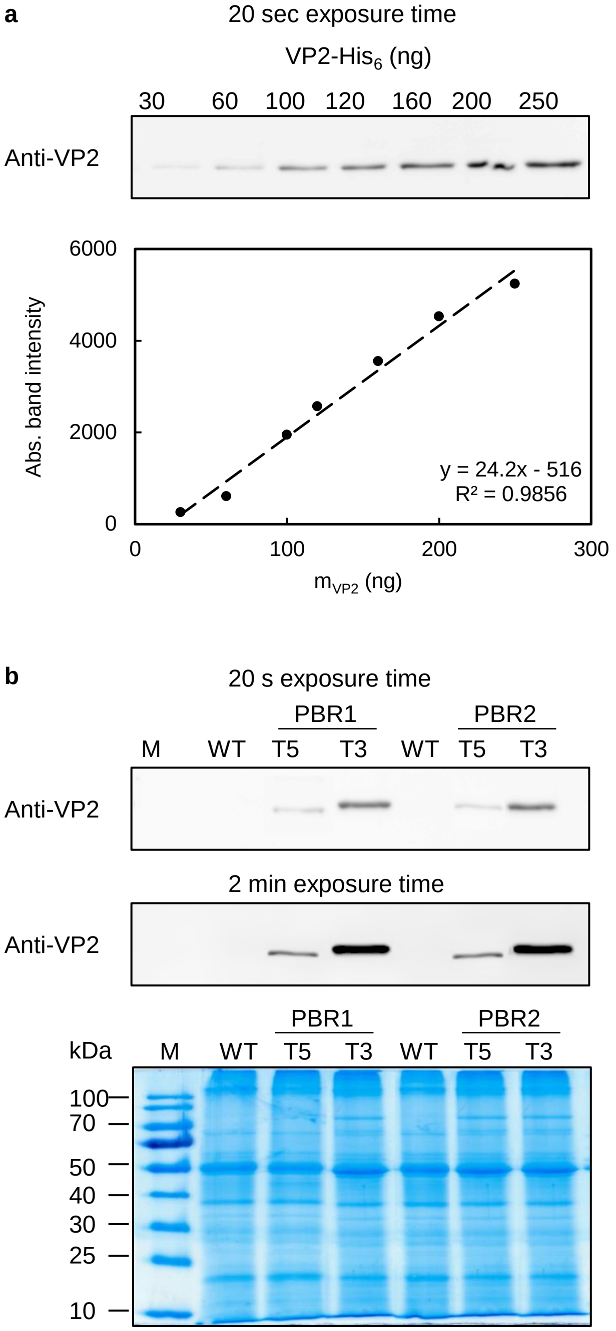

Figure S9 Exemplary quantification of the VP2 yield in *N. oceanica* transformants by a standard curve of purified VP2-His$_6$ and anti-VP2 immunoblotting. a Different amounts (30-250 ng) of VP2-His$_6$ (51 kDa) were analyzed by immunoblotting using a monoclonal antibody against VP2 (20 sec exposure time). The absolute band intensities were determined by ImageJ and a standard curve was generated. **b** Detection of VP2 in total soluble protein (TSP) in WT, T5 and T3 (each PBR1 and 2) by anti-VP2 immunoblotting and calculation of the relative VP2 content in TSP, which was performed in three technical replicates, as summarized in Table 1. The Coomassie-stained gel served to verify equal protein loading (5 µg). Also after longer exposure of the immunoblot of 2 min no cross-reactivity was observed for the WT. The experiments of **a** and **b** were carried out in parallel

Figure S10

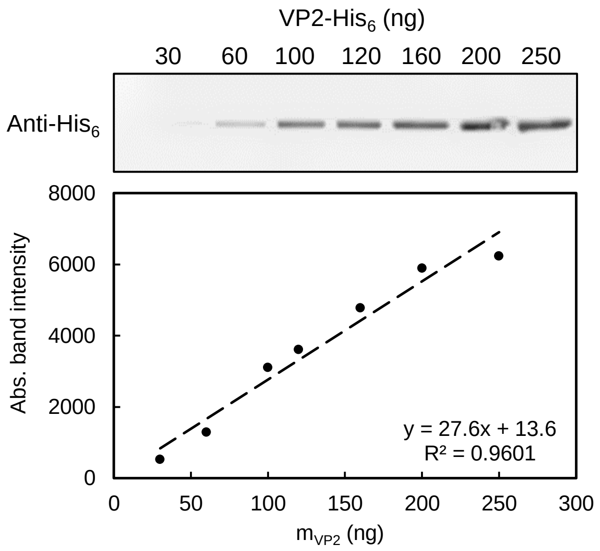

Figure S10 Confirmation of the VP2 yield by His_6–specific immunoblotting. Instead of the VP2-specific monoclonal antibody (Fig. S9), an anti-His_6-specific antibody was used here to generate a 2nd standard curve of different amounts of VP2-His_6 (30 to 250 ng, 51 kDa, 20 sec exposure time). The absolute band intensities were determined by ImageJ. For the T3 and T5 transformants, the VP2 content was re-determined at Day 20; the values differed by <0.2 % compared to anti-VP2-specific immunoblotting (Table 1)

Figure S11

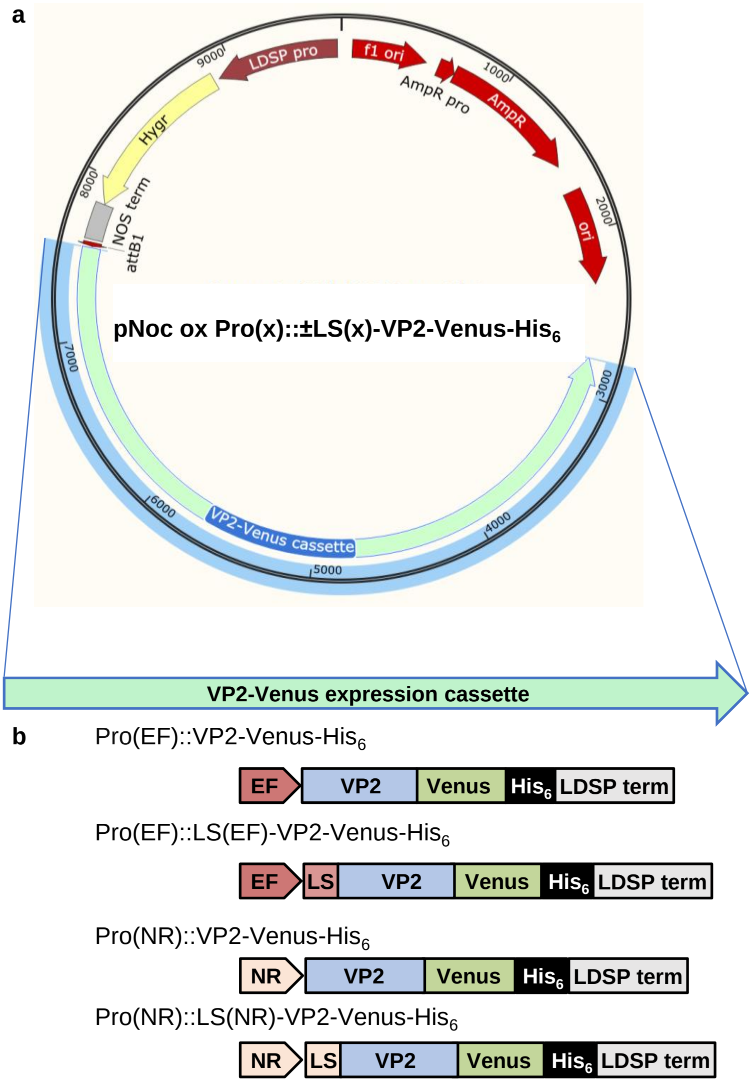

Figure S11 Vector map for the expression of *VP2-Venus* in *N. oceanica.* a One representative vector diagram is shown, here with the expression cassette of VP2-Venus (green). The vector map indicates the cassette of the hygromycin resistance gene (*HygR*, yellow) driven by the endogenous lipid droplet surface protein promoter (LDSP pro, brown) and the terminator of nopaline synthase (NOS term, grey). The details of the original vector pNoc ox are described in Zienkiewicz et al. (2017), and all vector sequences are listed in Data S1. **b** Four different versions of the expression cassette are depicted and differ in promoter (EF or NR) and LS identity and presence. Constant elements are the *VP2* CDS (blue) located upstream of *Venus* (green), followed by a C-terminal his_6 tag (black), and the terminator of lipid droplet surface protein (LDSP term, grey).

Figure S12

a

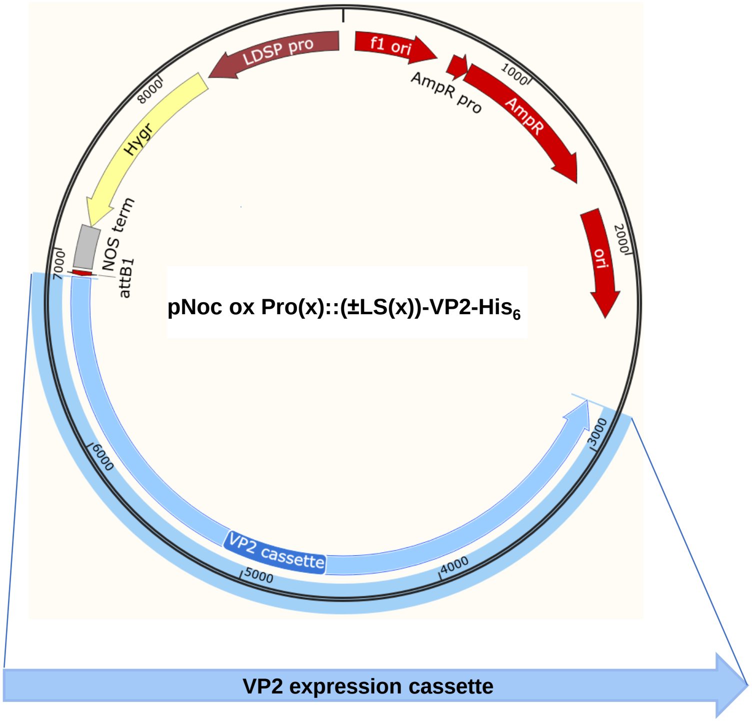

b Pro(EF)::VP2-His$_6$

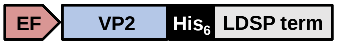

Pro(EF)::LS(EF)-VP2-His$_6$

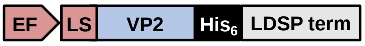

Figure S12 Vector map for the expression of reporter-free *VP2* in *N. oceanica*. a One representative vector diagram is shown, here with the expression cassette of VP2-His$_6$ (blue). **b** Two versions of the expression cassette with the EF promoter are depicted and differ in LS presence. Constant elements are the *VP2* CDS (blue) located upstream of *Venus* (green), followed by a C-terminal his$_6$ tag (black), and the terminator of lipid droplet surface protein (LDSP term, grey). For further details see legend of Figure S11.

Table S1 Codon usage table for *N. oceanica* created from the codons of 75 highly expressed genes (21,044 codons in total) under standard growth conditions (Vieler et al. 2012).

**Codon**	Frequency per 1000	Codon	Frequency per 1000
**Starting with U**		**Starting with A**	
UUU	17.4	AUU	15.7
UUC	21.9	AUC	20.9
UUA	3.5	AUA	3.0
UUG	29.3	AUG	29.1
UCU	8.8	ACU	7.6
UCC	16.5	ACC	18.0
UCA	4.1	ACA	7.8
UCG	13.5	ACG	18.7
UAU	9.5	AAU	11.9
UAC	17.8	AAC	17.8
UAA	1.5	AAA	12.9
UAG	1.1	AAG	44.2
UGU	4.1	AGU	7.4
UGC	7.2	AGC	15.7
UGA	0.7	AGA	4.5
UGG	13.5	AGG	11.4
**Starting with C**		**Starting with G**	
CUU	10.1	GUU	7.2
CUC	19.7	GUC	21.5
CUA	2.7	GUA	5.9
CUG	9.8	GUG	37.6
CCU	12.6	GCU	16.7
CCC	19.8	GCC	35.8
CCA	6.2	GCA	13.7
CCG	11.8	GCG	27.4
CAU	8.9	GAU	23.0
CAC	12.6	GAC	30.2
CAA	11.8	GAA	19.6
CAG	22.8	GAG	54.2
CGU	8.8	GGU	13.6
CGC	13.3	GGC	28.7
CGA	8.1	GGA	18.4
CGG	10.3	GGG	30.3

Table S2 Primers used for subcloning, detection of the expression vectors, and qRT-PCR analyses. Restriction sites are underlined. Fusions of VP2-Venus were generated by subcloning *VP2* upstream of the *Venus* reporter gene in the four pNoc ox expression vectors generated previously. Abbr.: fw, forward; rv, reverse.

Purpose	Construct / Target	Primer sequence (5‘-3‘)
Subcloning of VP2 into pNoc ox	Pro(EF):: (±LS(EF))-VP2-His_6	fw: AAA<u>CAATTG</u>ATGAACACGAACAAAGCCAC AGCG<br>rv: AAA<u>ACGCGT</u>TCAGTGATGGTGATGGTGATGGCCGT TCCAGCCTTCGAAGTGGGAAGGTCGGAG
Detection of expression vectors	Pro(EF)::(±LS(EF))-VP2-Venus-His_6	fw: AACACCTCTGGCTCATTC<br>rv: TGGCCGTTTACGTCG
	Pro(NR)::(±LS(NR))-VP2-Venus-His_6	fw: ATGACTGACCCGACTCTG<br>rv: TGGCCGTTTACGTCG
	Pro(EF):: (±LS(EF))-VP2-His_6	fw: AACACCTCTGGCTCA TTC<br>rv: CCTGCGGAACATTCTTAC
qRT-PCR	ACT2	fw: ACCTTCTACAACGAGCTGC<br>rv: GAACGTCTCAAACATAATCTGG
	Venus	fw: GCCGAAGTGAAGTTTGAGG<br>rv: GTCCGCGGTGATATACACG
	VP2	fw: CGAGTTGCTCAAGAACATGG<br>rv: GAAGGTCGGAGGAAAAGTCC
Southern blotting	HygR	fw: AAAGTTCGACAGCGTCTCC<br>rv: GCGTCGGTTTCCACTATCG
	VP2	fw: CAAAGCCACAGCGACGTACC<br>rv: AGACCCTTGTGCGTTCCTTG

Table S3: Backbone vector features common to the six expression vectors.

Name	Start	End	Size (bp)	Description	Type	Color code
f1 ori	69	524	456	f1 bacteriophage origin of replication	rep_origin	red
Amp^R pro	602	706	105	Promoter ampicillin gene	promoter	red
Amp^R	707	1567	861	Ampicillin resistance gene: beta-lactamase from *E.coli* (bla); confers resistance to ampicillin, carbenicillin, and related antibiotics	CDS	red
ori	1738	2326	589	High-copy-number ColE1/pMB1/pBR322/pUC origin of replication	rep_origin	red
LDSP term	2785	4452	1668	Terminator of lipid droplet surface protein		grey
attB1	7613	7637	25	mutant version of attB1; recombination site for the Gateway BP reaction	protein_bind	red
NOS term (complement)	7655	7880	226	Terminator of nopaline synthase gene	terminator	grey
HygR (complement)	7881	8906	1026	Hygromycin resistance gene: aminoglycoside phosphotransferase from *E.coli* (aph(4)-Ia); confers resistance to hygromycin	CDS	yellow
LDSP pro (complement)	8940	767	744	Lipid droplet surface protein	promoter	brown

Table S4 Alternative promoters for expression of VP2-Venus-His_6 and VP2-His_6

Promoter name	Vector name	Start	End	Size	Description	Color code
EF	Pro(EF)::VP2-Venus-His_6	6659	7560	992	Constitutive promoter of endogenous elongation factor gene	light red
	Pro(EF)::LS(EF)-VP2-Venus-His_6	6611	7602			
	Pro(EF)::VP2-His_6	5837	6828			
	Pro(EF)::LS(EF)-VP2-His_6	5879	6870			
NR	Pro(NR)::VP2-Venus-His_6	6569	7372	804	Inducible promoter of endogenous nitrate reductase gene	light red
	Pro(NR)::LS(NR)-VP2-Venus-His_6	6597	7400			

Table S5 Alternative CDS in different expression constructs

Gene name	Vector name	Start	End	Size	Description	Color code
LS(EF)	Pro(EF)::LS(EF)-VP2-Venus-His_6	6568	6610	43	The first 14 N-terminal amino acids of the elongation factor (EF) gene referred to as leader sequence (LS)	light red
	Pro(EF)::LS(EF)-VP2-His_6	5836	5878			
LS(NR)	Pro(NR)::LS(NR)-VP2-Venus-His_6	6569	6611	43	The first 14 N-terminal amino acids of the nitrate reductase (NR) gene referred to as leader sequence (LS)	light red
VP2	Pro(x)::(±LS(x))-VP2-Venus-His_6	5231	6562	1332	Viral surface protein (VP2) of infectious pancreatic necrosis virus (IPNV)	blue
	Pro(EF)::(±LS(EF))-VP2-His_6	4505	5840			
Venus	Pro(x)::(±LS(x))-VP2-Venus-His_6	4505	5221	717	Reporter gene	green
His_6	Pro(EF):: LS(EF)-VP2-Venus-His_6	4478	4495	18	Hexahistidine tag	black

Table S6 Amino acid sequences of alternative CDS in different expression constructs

CDS name	Amino acid sequence	Molecular weight (kDa)	Size (aa)	Color code
LS(EF)	MGKEKTHVNLVVIG	1.5	14	light red
LS(NR)	MAFKLSPQVPTAPE	1.5	14	light red
VP2	MNTNKATATYLKSIMLPETGPASIPDDITERHILKQ ETSSYNLEVSESGSGILVCFPGAPGSRIGAHYRW NANQTGLEFDQWLETSQDLKKAFNYGRLISRKYDI QSSTLPAGLYALNGTLNAATFEGSLSEVESLTYNS LMSLTTNPQDKVNNQLVTKGVTVLNLPTGFDKPY VRLEDETPQGLQSMNGAKMRCTAAIAPRRYEIDL PSQRLPPVTATGALTTLYEGNADIVNSTTVTGDINF SLTEQPAVETKFDFQLDFMGLDNDVPVVTVVSSV LATNDNYRGVSAKMTQSIPTENITKPITRVKLSYKI NQQTAIGNVATLGTMGPASVSFSSGNGNVPGVLR PITLVAYEKMTPLSILTVAGVSNYELIPNPELLKNMV TRYGKYDPEGLNYAKMILSHREELDIRTVWRTEEY KERTRVFNEITDFSSDLPTSKAQLGSGHHHHHH	48.8	444	blue
Venus	MVSKGEELFTGVVPILVELDGDVNGHKFSVSGEG EGDATYGKLTLKLICTTGKLPVPWPTLVTTLGYGL QCFARYPDHMKQHDFFKSAMPEGYVQERTIFFKD DGNYKTRAEVKFEGDTLVNRIELKGIDFKEDGNIL GHKLEYNYNSHNVYITADKQKNGIKANFKIRHNIED GGVQLADHYQQNTPIGDGPVLLPDNHYLSYQSKL SKDPNEKRDHMVLLEFVTAAGITLGMDELYK	26.9	239	green
His_6	HHHHHH	0.85	6	black
