# Supplementary material for: Production of a viral surface protein in Nannochloropsis oceanica for fish vaccination against infectious pancreatic necrosis virus
Source: Appl Microbiol Biotechnol. 2022 Sep 7;106(19-20):6535–49. doi: 10.1007/s00253-022-12106-7 (PMC9449291; doi:10.1007/s00253-022-12106-7)
Supplement: Supplementary file 2 — Supplementary file2 (PDF 124 KB) [file 253_2022_12106_MOESM2_ESM.pdf]

Data S1 Nucleotide sequence of the expression vector Pro(EF)::LS(EF)-VP2-Venus-His$_6$ and related vector information. The nucleotide sequence of one plasmid [Pro(EF)::LS(EF)-VP2-Venus-His$_6$] is representatively given below with the color code corresponding to that of the vector maps (Fig. S11 and 12), which were produced using SnapGene (www.Snapgene.com; v6.0.5). Primers are listed in Table S2. The sequences of four Venus-tagged VP2 constructs [Pro(x)::(±LS(x))-VP2-Venus-His$_6$] and two reporter-free VP2 constructs [Pro(x)::(±LS(x))-VP2-His$_6$] are described below. The common components of the backbone vector are listed in Table S3. Vector-specific features (e.g., promoters, genes of interest and amino acid positions) are listed in Tables S4-S6

```
   1 ggcgtaatag cgaagaggcc cgcaccgatc gcccttccca acagttgcgc agcctgaatg gcgaatggac
  71 gcgccctgta gcggcgcatt aagcgcggcg ggtgtggtgg ttacgcgcag cgtgaccgct acacttgcca
 141 gcgccctagc gcccgctcct ttcgctttct tcccttcctt tctcgccacg ttcgccggct ttccccgtca
 211 agctctaaat cgggggctcc ctttagggtt ccgatttagt gctttacggc acctcgaccc caaaaaactt
 281 gattagggtg atggttcacg tagtgggcca tcgccctgat agacggtttt tcgccctttg acgttggagt
 351 ccacgttctt taatagtgga ctcttgttcc aaactggaac aacactcaac cctatctcgg tctattcttt
 421 tgatttataa gggattttgc cgatttcggc ctattggtta aaaaatgagc tgatttaaca aaaatttaac
 491 gcgaatttta acaaaatatt aacgcttaca atttcctgat gcggtatttt ctccttacgc atctgtgcgg
 561 tatttcacac cgcatcaggt ggcacttttc ggggaaatgt gcgcggaacc cctatttgtt tatttttcta
 631 aatacattca aatatgtatc cgctcatgag acaataaccc tgataaatgc ttcaataata ttgaaaaagg
 701 aagagtATGa gtattcaaca tttccgtgtc gcccttattc ccttttttgc ggcattttgc cttcctgttt
 771 ttgctcaccc agaaacgctg gtgaaagtaa aagatgctga agatcagttg ggtgcacgag tgggttacat
 841 cgaactggat ctcaacagcg gtaagatcct tgagagtttt cgccccgaag aacgttttcc aatgatgagc
 911 acttttaaag ttctgctatg tggcgcggta ttatcccgta ttgacgccgg gcaagagcaa ctcggtcgcc
 981 gcatacacta ttctcagaat gacttggttg agtactcacc agtcacagaa aagcatctta cggatggcat
1051 gacagtaaga gaattatgca gtgctgccat aaccatgagt gataacactg cggccaactt acttctgaca
1121 acgatcggag gaccgaagga gctaaccgct tttttgcaca acatggggga tcatgtaact cgccttgatc
1191 gttgggaacc ggagctgaat gaagccatac caaacgacga gcgtgacacc acgatgcctg tagcaatggc
1261 aacaacgttg cgcaaactat taactggcga actacttact ctagcttccc ggcaacaatt aatagactgg
1331 atggaggcgg ataaagttgc aggaccactt ctgcgctcgg cccttccggc tggctggttt attgctgata
1401 aatctggagc cggtgagcgt gggtctcgcg gtatcattgc agcactgggg ccagatggta agccctcccg
1471 tatcgtagtt atctacacga cggggagtca ggcaactatg gatgaacgaa atagacagat cgctgagata
1541 ggtgcctcac tgattaagca ttggtaactg tcagaccaag tttactcata tatactttag attgatttaa
1611 aacttcattt ttaatttaaa aggatctagg tgaagatcct ttttgataat ctcatgacca aaatccctta
1681 acgtgagttt tcgttccact gagcgtcaga ccccgtagaa aagatcaaag gatcttcttg agatcctttt
1751 tttctgcgcg taatctgctg cttgcaaaca aaaaaaccac cgctaccagc ggtggtttgt ttgccggatc
1821 aagagctacc aactcttttt ccgaaggtaa ctggcttcag cagagcgcag ataccaaata ctgttcttct
1891 agtgtagccg tagttaggcc accacttcaa gaactctgta gcaccgccta catacctcgc tctgctaatc
1961 ctgttaccag tggctgctgc cagtggcgat aagtcgtgtc ttaccgggtt ggactcaaga cgatagttac
2031 cggataaggc gcagcggtcg ggctgaacgg ggggttcgtg cacacagccc agcttggagc gaacgaccta
2101 caccgaactg agatacctac agcgtgagct atgagaaagc gccacgcttc ccgaagggag aaaggcggac
2171 aggtatccgg taagcggcag ggtcggaaca ggagagcgca cgagggagct tccaggggga aacgcctggt
2241 atctttatag tcctgtcggg tttcgccacc tctgacttga gcgtcgattt ttgtgatgct cgtcaggggg
2311 gcggagccta tggaaaaacg ccagcaacgc ggccttttta cggttcctgg ccttttgctg gccttttgct
2381 cacatgttct ttcctgcgtt atcccctgat tctgtggata accgtattac cgcctttgag tgagctgata
2451 ccgctcgccg cagccgaacg accgagcgca gcgagtcagt gagcgaggaa gcggaagagc gcccaatacg
2521 caaaccgcct ctccccgcgc gttggccgat tcattaatgc agcctagtaa catagatgac accgcgcgcg
2591 ataatttatc ctagtttgcg cgctatattt tgttttctat cgcgtattaa atgtataatt gcgggactct
2661 aatcataaaa acccatctca taaataacgt catgcattac atgttaatta ttacatgctt aacgtaattc
2731 aacagaaatt atatgataat catcgcaaga ccggcaacag gattcaatct taaggtgatg ctgttgctct
2801 ttccaatgat gatgacagca ctggcacgaa tctcgtcgat cgagttgtaa aggcaaggga ggagggcaga
2871 gtagtccaag agggtgaaga cgcggctgca gtcttcgttc aggaggtgca gcagcagccg cagcagatat
2941 cagccgcaac agcaacagca acagcaattt tatcgaagat ggaagcagag gcagcaagct atatttctac
3011 acacacttat tctccgtctc ctcccctcc tcctccacct caagaagtag tcgattttag gttatctgac
3081 gcccgggaat cctcggtttg tctgcatcac ctcactctcc actctcctat cccttgcgcg gtccttcaca
3151 aagagcttgc ttcctttgca acgcaagact tgataaaagg agaggacgat gcggaggagg aggcgaagag
3221 aggaggggga acaaagagac aaaggaagaa gaagagcaag gggaagaagg tggggaggaa aagagggatg
3291 gttcccctga tagaaggagt gacacgatgg gtggaagagt tttggggaaa ggcggtgaga gcaacggcta
3361 atagtttagc cagctccaat gacatcacat cgtcttcctc ctcctccgcc tcgtcttctg tctgtacttg
3431 cccttgtgct gttgacacag gtgccgctgc tcctgctgct cctgcttctg ttgctgtttg cggcgaggta
3501 ggggagaagg aaaaggaccg aatcgaggct ctgataaatg aggtaggtga gctgcgttcg cagctagcgg
3571 atttcttaga agagatgcga ggagggaggg agggagggag ggaggagtgg gatcgatgat ggttgcaccg
3641 atggattgcg agggagatgt gccatgcgat ctagtgtaaa gtagaagata aacaggagaa gatgatgggt
3711 gagagaaggc gggggggagg tggcggttga tttttcggga aagattacaa aataaaaagg ctgcatgtac
3781 atgcacgcgg ggtaggtgat aacaaagaac gagacgatat cacctcttct gtttccacga taaaaataga
3851 ctgctcattt cttcgtcgtc ttcatcgtct gctttttctg cttcgcctct gtctggggtc tgaaaccact
3921 acacacacac acaacactcg tactcccact ttcacaaaag cgtaagctca ccggcttttc ttacacgtac
3991 attttagtgg atcccatcac gccactacca cgcccgcggg ggatggaacg gaggggagag agagaagggg
4061 gaagcatgga tgaatgagac attgagggaa aggaggggag ggagcagtcc atcagggcgc tacctctctt
4131 gtcccccaaa ccctgttgag ccgttcaaca tgtttcatgt ttcctccctc ccccttccc tccctggcct
4201 ttccgcggag ccattcaagt gacgtctgga ccgcaccgta acaaaatcgt ttctatgggg ggtttgtttg
4271 acaaccacga agagcagcgt ttttaaaaaa aaaagcgggc aagccctctc accctcactc atgcccatcc
4341 tcctcctctc ctgcggaaca ttcttacaaa aggcgtaact cgacgacaac tcaaagaacg acaaacatca
4411 atcccaaaaa aaaaatctct actcgtctct cttggatctt tcgagctcat cgcgactcac gcgttcagtg
4481 atggtgatgg tgatggccgc ttcctttgta caactcatcc atcccaagcg tgatacctgc tgccgtgacg
4551 aattcgagca acaccatgtg gtcgcgcttc tcgttagggt ccttggagag cttgctttgg tagctcaggt
4621 aatggttgtc cggaagcaac acgggtccgt cgccaatggg cgtattttgc tgataatgat ccgccagctg
4691 cactccccca tcctcaatat tgtgccggat cttgaagtta gccttgatgc cattcttttg tttgtccgcg
4761 gtgatataca cgttatgact attgtagttg tactccaatt tgtgtcccaa aatgttccca tcctccttga
```

4831 agtcgatccc cttcaattca attcggttga ccaaggtatc cccctcaaac ttcacttcgg cccgtgtctt
4901 gtaatttccg tcatccttaa agaagatcgt gcgctcctgg acatatcctt cgggcatggc cgacttaaag
4971 aagtcgtgct gcttcatgtg gtcagggtac ctggcaaagc actggagccc ataaccaagc gtggtgacca
5041 gagtaggcca cggcactggg agtttcccgg ttgtacagat aagcttcagg gtcagcttgc cgtaggtggc
5111 atcgccctcg ccctcgccgg acacgctgaa cttgtggccg tttacgtcgc cgtccagctc gaccaggatg
5181 ggcaccaccc cggtgaacag ctcctcgccc ttgctcac**CA** **T**gccgctccc caattgagcc ttcgaagtgg
5251 gaaggtcgga ggaaaagtcc gtaatctcgt taaagaccct tgtgcgttcc ttgtactcct cagtacgcca
5321 caccgtgcgg atatccagct cttctcggtg agacaagatc atcttggcgt aattcaagcc ctcgggatcg
5391 tacttcccgt aacgagtcac catgttcttg agcaactcgg gattggggat aagctcgtaa ttcgacaccc
5461 ctgcgacggt caggatggag agcggagtca tcttctcata cgcgaccagc gtaatcggcc gcaagacccc
5531 aggcacgttc ccattgccag aggagaagga cacggaggcc ggacccatgg tacccaacgt ggctacattt
5601 ccgatcgcgg tctgctgatt gatcttgtag gacagcttca ccctagtgat gggtttcgtg atgttctccg
5671 ttggaatact ctgggtcatc ttggccgata cccctcggta gttgtcattc gttgcaagca cgctagaaac
5741 gaccgtaacg acaggcacat cattgtccaa gcccatgaag tcgagctgga agtcgaactt cgtctccaca
5811 gcgggctgtt cggtgaggct gaagttgata tcgcccgtaa cggttgtgga gttcacgatg tcggcattgc
5881 cctcatagag ggtcgtcaac gctccggtag cagtcacggg aggcaatcgt tgggatggca gatcgatctc
5951 gtaccgccga ggtgcaatgg cagctgtaca gcgcattttc gccccgttca tcgactgcaa gccttgcggt
6021 gtttcatcct ccaagcgcac atagggctta tcgaaccctg tggggagatt caagacggtc acgcctttgg
6091 tcaccagctg gttgttgacc ttgtcctggg gattggtggt aagcgacatc aagctgttgt aggtcaagga
6161 ctcgacctcc gacaaggatc cctcaaaggt tgcggcgttg agagttccgt tgagggcata caagcccgca
6231 ggaagcgtcg agctttgaat gtcgtacttg cgagagatca gccttccata gttaaaggct ttcttgagat
6301 cctggctcgt ctccagccac tggtcgaact cgagccctgt ttggttggca ttccagcgat aatgcgcccc
6371 aatacgacta ccaggagcac ccggaaagca caccaaaatg ccgcttccag attcgctcac ctccaggttg
6441 taactgctgg tttcttgctt caagatgtgc ctctccgtaa tgtcatccgg aatactcgct ggcccggttt
6511 caggcaacat gatgctcttc aggtacgtcg ctgtggcttt gttcgtgtt**C** **AT**caattggc caatcacgac
6581 caggttcacg tgggtcttct cctttcc**CAT** tgttacgaag tgagggttga ggggtggggg tgctgtgctc
6651 gtggtggatg ggtgaggagg agagggagaa tgagccagag gtgtttatca aagtcctatg aacaaaatgc
6721 cggcgcgggc cgtgcgtgag gcgcgtacat gcccatcgat gccacgaccc tggccggtgg ctggcagggc
6791 gaggtggtgc agcgccggca gtgcagcaaa gaagccacgc cgccgtccgg cccaacaacg agaagccttg
6861 cgtctcatgc tgaacgaggc atgtcccacc tgcgcgccga aacaggtgag tgtaattccc atgagggata
6931 cgtattgttg tgacgggctg cgcggcaaag gcacgcgcaa accgcagcca tcccctgctt atcttgtttt
7001 cttctttcgc tgtgctcaag gcttctgccg accacgcatt ccactaccac atgcggggtt cgggccactc
7071 agcgcgcgac taacttggcc cgacacctgc atacatgtgc aagaggagtg gtggcgggtg ggtgaatgag
7141 ctgtgcgcag aagcgcatcg taaatcgact aagagagaag tgatgcacgc ccttcttccg tgtcgccgca
7211 tccgcagcca cacacctcca cggaacaggg tctgcctccc gcgacaccgg tcaacccatg cacccaaaga
7281 tgtcagaaga cagctgagaa tccagccatc aatcacgagg tgatcaaaag catgcagcaa aagcgaccac
7351 tacttaaaga tgaggagtca cacgccgcct ccacccacga aagccaagaa tatcgggagc cacccgtgcc
7421 ggcgacggtc taaaccgctc ttgctgacga ccgtaataaa atgacgaggt tgaccgtaat ctggcccatt
7491 gggcttcgtt ttgcaagtgc tgcctgccgc tcccgcgccg cggaagtcat tttgtatact ttacgctatg
7561 tctttatttc ctttgacgac tactagctac catgtagcta tagcggccgc ggagcctgct tttttgtaca
7631 aacttgttga tatcgcctgc aggtcactgg attttggttt taggaattag aaattttatt gatagaagta
7701 ttttacaaat acaaatacat actaagggtt tcttatatgc tcaacacatg agcgaaaccc tataagaacc
7771 ctaattccct tatctgggaa ctactcacac attattctgg agaaaaatag agagagatag atttgtagag
7841 agagactggt gatttttgcg gactccggtc ggcatctact ctattccttt gccctcggac gagtgctggg
7911 gcgtcggttt ccactatcgg cgagtacttc tacacagcca tcggtccaga cggccgcgct tctgcgggcg
7981 atttgtgtac gcccgacagt cccggctccg gatcggacga ttgcgtcgca tcgaccctgc gcccaagctg
8051 catcatcgaa attgccgtca accaagctct gatagagttg gtcaagacca atgcggagca tatacgcccg
8121 gagccgcggc gatcctgcaa gctccggatg cctccgctcg aagtagcgcg tctgctgctc catacaagcc
8191 aaccacggcc tccagaagaa gatgttggcg acctcgtatt gggaatcccc gaacatcgcc tcgctccagt
8261 caatgaccgc tgttatgcgg ccattgtccg tcaggacatt gttggagccg aaatccgcgt gcacgaggtg
8331 ccggacttcg gggcagtcct cggcccaaag catcagctca tcgagagcct gcgcgacgga cgcactgacg
8401 gtgtcgtcca tcacagtttg ccagtgatac acatggggat cagcaatcgc gcatatgaaa tcacgccatg
8471 tagtgtattg accgattcct tgcggtccga atggccgaa cccgctcgtc tggctaagat cggccgcagc
8541 gatcgcatcc atggcctccg cgaccggctg cagaacagcg ggcagttcgg tttcaggcag gtcttgcaac
8611 gtgacaccct gtgcacggcg ggagatgcaa taggtcaggc tctcgctgaa ttccccaatg tcaagcactt
8681 ccggaatcgg gagcgcggcc gatgcaaagt gccgataaac ataacgatct ttgtagaaac catcggcgca
8751 gctatttacc cgcaggacat atccacgccc tcctacatcg aagctgaaag cacgagattc ttcgccctcc
8821 gagagctgca tcaggtcgga gacgctgtcg aacttttcga tcagaaactt ctcgacagac gtcgcggtga
8891 gttcaggctt ttt**CAT**atct tattgccccc cggggccctc ctgtgttgat gcgggctgag attggtggtg
8961 gtctatcacg aatatgtgtg aggggtaagt gcggtgtttt gcgtgagatt ttagaatatt gccccgcccc
9031 ggggcaggcc ggcgtggcgg aacaaccagg cacacgagcg cgaatggtga taccgacgga gtcaaaactt
9101 tgtgacaagt agctgcacca tgggcagtgg tgagctttca gacgtggtat cactgtccac tagttcacac
9171 acagaatgcg tgtccaaaag gtctagagcc gtctcgcttg cgtctctccg tcgaagaaca gtgaagaggc
9241 tcgtcacgtc gaccagacga cgggaggctg gtcaccatcg cagatgtctc ccacaaagca gcacggcaac
9311 tcctactcct tcacacaatg gaagaaaagg tggtctgatg gttctcagtg gaaaagaacg atatcaggct
9381 gaaaaaaatg atctgcaggc tccagattcc tgaatcacgt cgactgtgac gaagcaaacc gcgtcgaaca
9451 acatcggtca tgccaacggg tctcgtctct cgagcccttt tggcggcgac taagaagtat gaagcttcag
9521 gccgcaacgc gcgacacagc gttttgtgtg gtgggcctcg gcattgctct ttgcatggcc cagcgtgatt
9591 agtgcgtgga ttttaagccc gagaccgaag gattgcgaca tgtgcctggc tgtataactc acgcttgctg
9661 ctacgctcgc ctcctcctcc atccactcca tcttagagac ctacct
